# Single Transmembrane Peptide DinQ Modulates Membrane-Dependent Activities

**DOI:** 10.1371/journal.pgen.1003260

**Published:** 2013-02-07

**Authors:** Ragnhild Weel-Sneve, Knut Ivan Kristiansen, Ingvild Odsbu, Bjørn Dalhus, James Booth, Torbjørn Rognes, Kirsten Skarstad, Magnar Bjørås

**Affiliations:** 1 Centre for Molecular Biology and Neuroscience (CMBN), University of Oslo and Oslo University Hospital, Rikshospitalet, Oslo, Norway; 2 Department of Microbiology, University of Oslo and Oslo University Hospital, Rikshospitalet, Oslo, Norway; 3 Department of Cell Biology, Institute for Cancer Research, University of Oslo and Oslo University Hospital, Radiumhospitalet, Oslo, Norway; 4 Department of Biochemistry, University of Oslo and Oslo University Hospital, Rikshospitalet, Oslo, Norway; Baylor College of Medicine, United States of America

## Abstract

The functions of several SOS regulated genes in *Escherichia coli* are still unknown, including *dinQ*. In this work we characterize *dinQ* and two small RNAs, *agrA* and *agrB*, with antisense complementarity to *dinQ*. Northern analysis revealed five *dinQ* transcripts, but only one transcript (+44) is actively translated. The +44 *dinQ* transcript translates into a toxic single transmembrane peptide localized in the inner membrane. *AgrB* regulates *dinQ* RNA by RNA interference to counteract DinQ toxicity. Thus the *dinQ-agr* locus shows the classical features of a type I TA system and has many similarities to the *tisB*-*istR* locus. DinQ overexpression depolarizes the cell membrane and decreases the intracellular ATP concentration, demonstrating that DinQ can modulate membrane-dependent processes. Augmented DinQ strongly inhibits marker transfer by Hfr conjugation, indicating a role in recombination. Furthermore, DinQ affects transformation of nucleoid morphology in response to UV damage. We hypothesize that DinQ is a transmembrane peptide that modulates membrane-dependent activities such as nucleoid compaction and recombination.

## Author Summary

Exposure of the bacterium *Escherichia coli* to DNA damaging agents induces the SOS response, which up-regulates gene functions involved in numerous cellular processes such as DNA repair, cell division, and replication. Most of the SOS regulated genes in *E. coli* have been characterized, but still there are several genes of unknown function. One of these uncharacterized genes is *dinQ*. In this work we characterize *dinQ* and two novel small RNAs, *agrA* and *agrB*, that regulate expression of *dinQ*. The DinQ peptide is localized in the inner membrane as a single transmembrane peptide of 27 amino acids. Small proteins of less than 50 amino acids are important in cellular processes such as regulation, signalling, and antibacterial action. Here we demonstrate that DinQ modulates recombination and transformation of nucleoid morphology in response to UV damage. Our results provide new insights into small hydrophobic peptides that could regulate important DNA metabolic processes dependent on the inner membrane of the cell.

## Introduction

Exposure of *E. coli* to DNA damaging agents induces the SOS response, which is under control of the RecA and LexA regulatory proteins. The SOS response upregulates gene functions involved in numerous cellular processes such as nucleotide excision repair (NER), UV induced mutagenesis, recombination, inhibition of cell division and replication. The LexA repressor downregulates more than 50 SOS genes by binding to the operator sequence in their promoter regions [1], [2]. SOS inducers (e.g. UV) cause replication blocks and generate RecA/ssDNA nucleoprotein filaments that mediate auto-proteolysis of the LexA repressor.

Both NER and recombination are required to maintain DNA integrity. NER repairs numerous lesions introducing helical distortions, in which UvrA, UvrB and UvrC work in sequential steps to recognize and remove the lesion. The RecBCD complex is the major component for initiation of recombinational repair (RR) of DNA double strand breaks (DSBs) by processing a blunt dsDNA end into a dsDNA molecule possessing a 3′-terminated ssDNA tail. As part of this process RecBCD mediates RecA filamentation required for presynaptic processing of dsDNA ends.

Most of the characterized LexA regulated genes play important roles in the physiology of *E. coli*, but there are still several genes of unknown function. One of these uncharacterized genes is *dinQ*, which is located in the 823 bp region between *arsR* and *gor*, 78.58 min on the *E. coli* chromosome. *DinQ* is predicted to encode an open reading frame (ORF) of 49 aa 139 nt downstream from a LexA operator sequence [1]. Small proteins of less than 50 amino acids are important in cellular processes such as regulation, signalling and antibacterial action [3]–[5]. More than 50 chromosomally encoded small proteins with a validated expression of less than 50 aa have been identified so far in *E. coli* [6]–[10]. Several of the newly discovered peptides are hydrophobic single transmembrane helices belonging to toxin-antitoxin systems.

In this work we characterize the *arsR*-*gor* intergenic region in which an endonucleolytic product of *dinQ* is translated into a small hydrophobic peptide of 27 aa. *DinQ* is under LexA control and antisense regulation by a novel small RNA, *agrB*. DinQ is localized in the inner membrane as a single transmembrane peptide that modulates nucleoid compaction and conjugal recombination.

## Results

### The *arsR*-*gor* intergenic region encodes two small RNAs, *agrA* and *agrB*, which are transcribed in the opposite direction to *dinQ*

**Figure 1:**
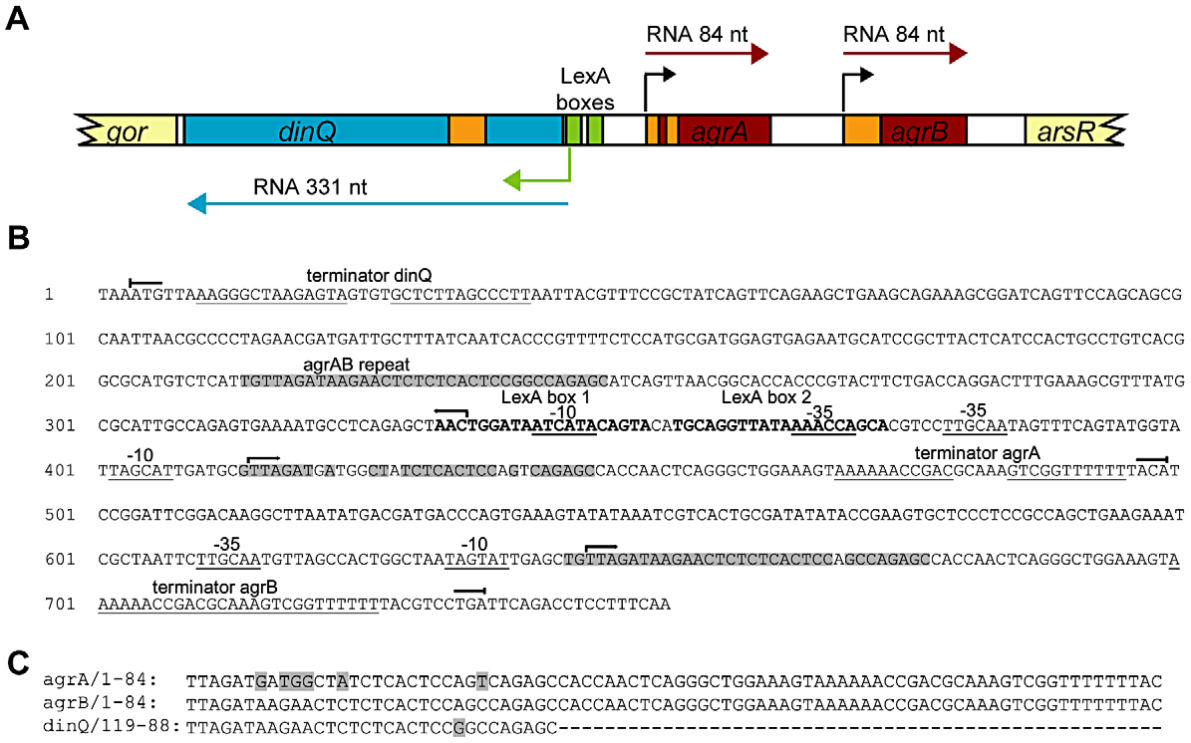
Overview of the *dinQ*/*agrAB* locus. (A) Genomic organization. The locus contains three genes originating from separate promoters; two constitutively expressed transcripts, *agrA* and *agrB* (red box), and one divergently transcribed gene, *dinQ* (blue box), which is regulated by LexA repressor binding sites (green box). Transcription initiation sites are indicated by color-coded arrows. Antisense sequences are annotated in orange. Flanking genes are indicated in light yellow. (B) Sequence of the full length *arsR-gor* intergenic region with hallmarks. Promoter elements, −10 and −35 and terminator sequences are underlined. LexA operator sequences in bold. Transcription start (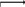) and stop (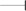) for *dinQ*, *agrA* and *agrB*. *AgrAB* repeat sequences are shadowed. (C) Alignment of *agrA/agrB* sequences antisense to *dinQ*. The *agrAB* repeat of *dinQ* is antisense to sequences in the *agrA* and *agrB* transcripts.

The SOS inducible *dinQ* gene in *E. coli* was identified in a search for new LexA regulated genes [1]. The *dinQ* gene was found in the 823 bp *arsR-gor* intergenic region (Figure 1A), encoding a ∼330 nt transcript with a putative LexA operator sequence (heterology index (HI) = 4.83) in the promoter region and two putative ORFs of 18 and 49 amino acids. No biological function, phenotype or significant homologies to proteins with known function were associated with DinQ [1]. Except for the *dinQ* gene, no other genes have been reported in the *arsR-gor* intergenic region. We performed a search for promoter and transcriptional terminator sequences in the *arsR-gor* intergenic region. As expected this search identified the *dinQ* LexA operator sequence identified earlier in a screen for LexA regulated genes in *E. coli* [1]. However, a second operator sequence for LexA (HI = 13.82) in close proximity to the first was identified (Figure 1A and 1B). Further, we identified putative −10 and −35 sequences corresponding to the *dinQ* promoter which overlaps both operator sequences, and a putative *dinQ* terminator sequence a few nucleotides downstream of the translational stop codon of the *gor* gene (Figure 1B). Finally, the sequence search identified two new small noncoding RNAs, termed *agrA* and *agrB* (*arsR*-*gor* region gene A and B, respectively), containing consensus like −10 and −35 sequences and rho independent terminator sequences (Figure 1A and 1B). *AgrA* and *agrB* are transcribed in the opposite direction of *dinQ* but encode no putative ORFs. Thirty-one nucleotides at the 5′ end of *agrA* and *agrB* show antisense complementarity to *dinQ* (Figure 1B and 1C). Twenty-five out of 31 nucleotides show complementarity to *agrA* while 30 out of 31 nucleotides show complementarity to *agrB*. This putative base pairing is indicative of possible RNA interference with the *dinQ* transcript. It thus appears that the *arsR-gor* region contain one protein coding gene, *dinQ*, and two small non-coding RNAs, *agrA* and *agrB*, with antisense complementarity to *dinQ*.

### Five *dinQ* transcripts

**Figure 2:**
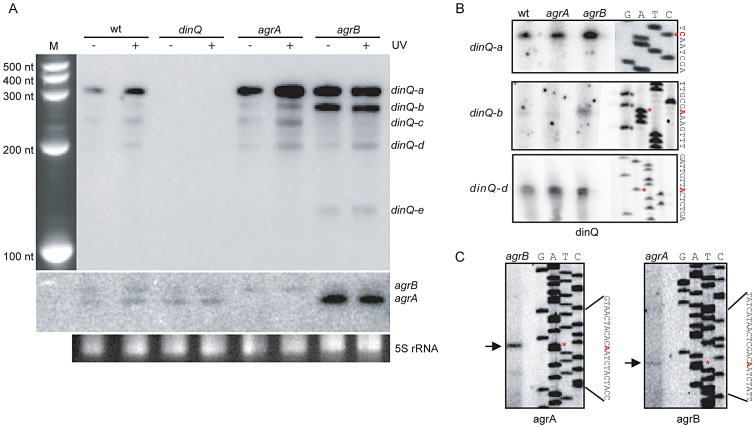
Expression pattern and transcription start of the *dinQ/agrAB* locus. (A) Northern analysis of *dinQ* and *agrAB* transcripts. A riboprobe specific to either *dinQ* (upper panel) or *agrAB* (lower panel) was hybridized to a northern blot with total RNA extracted from strains lacking *dinQ* (BK4040), *agrA* (BK4042) or *agrB* (BK4043) and compared to wt (AB1157) before and after UV exposure. Five *dinQ* transcripts assigned *dinQ-a, -b, -c, -d and -e* were detected. (B) A [^32^P]-labeled primer specific to *dinQ* was used in a primer extension assay to determine transcription initiation sites of the various *dinQ* transcripts. The primer extension assays were performed on total RNA extracted from wt (AB1157) and mutant strains *agrA* (BK4042) and *agrB* (BK4043). The base annotated for transcription initiation is indicated with an asterisk (red) for each transcript. Three primer extension products were detected that correlated in size to *dinQ-a, -b and -d* from 3′ mapping and the northern blot in (A). (C) A [^32^P]-labeled primer specific to *agrAB* was used in a primer extension assay to determine transcription initiation sites of *agrA* and *agrB*. Total RNA from mutant strain *agrB* (BK4043) was used to localize *agrA* transcription start and total RNA from mutant strain *agrA* (BK4042) was used to localize *agrB* transcription initiation, due to the transcripts sequence complementarity. The base annotated for transcription initiation is indicated with an asterisk (red) for each transcript.

To examine a potential role for the small non-coding RNAs *agrA* and *agrB* in regulating *dinQ*, three single mutants (*dinQ, agrA and agrB*), one double mutant (*agrAB*) and one triple mutant (*dinQ agrAB*) were generated (Table S1). To estimate the approximate size of the *dinQ*, *agrA* and *agrB* transcripts, northern blots with total RNA isolated from UV exposed (and unexposed) wild type and mutant strains (*dinQ*, *agrA* and *agrB*) were hybridized with radiolabeled riboprobes against the respective genes (Figure 2A). The *dinQ* probe generates five specific signals (*a*–*e*), in which the main transcript (*a*) migrates according to the expected size of full-length *dinQ*, ∼330 nt. *DinQ-b, -c, -d* and *-e* migrates as transcripts of about 290 nt, 250 nt, 200 nt and 130 nt, respectively, according to the size marker. All signals are absent in the *dinQ* mutant demonstrating that all five transcripts are derived from *dinQ*. The full-length *dinQ* product is 3- and 4.6-fold upregulated in the *agrA* and *agrB* mutants respectively under normal growth (without UV exposure). Notably, the *dinQ-b* signal is 4.8- and 3-fold upregulated in the *agrB* mutant as compared to the wild type and *agrA* mutant respectively. The *dinQ-c* product is not detectable in the *agrB* mutant. Further, the *dinQ* transcripts are induced in response to UV in wild type and *agrA* but not in *agrB*. These data indicate a regulatory mechanism by RNA interference, in which the *agrA* and *agrB* interfere differently with the *dinQ* transcript. Further, primer extension and 3′ mapping of *dinQ* RNA revealed transcript starts at 0, +44 and +125 corresponding to the estimated size of *dinQ-a*, *-b* and *–d*, respectively (Figure 2B). In agreement with the northern analysis we find that the +44 primer extension product (*dinQ-b*) is upregulated in the *agrB* mutant as compared to wild type and *agrA* mutant. The primer extension could not identify any products corresponding to *dinQ-c* or *-e*. The *agrAB* probes showed that the *agrB* transcript migrates slightly slower than the *agrA* transcript, and none of the transcripts were regulated in response to UV irradiation (Figure 2A, middle panel). The *agrA* transcript is upregulated 9.5 times in the unexposed *agrB* mutant as compared to wild type, indicating that the absence of *agrB* somehow promotes *agrA* RNA stability or transcription of *agrA*. Further, primer extension revealed that the sequence of transcription start was identical for the *agrA* and *agrB* genes (Figure 2C). 3′ mapping of *agrA* and *agrB* showed different transcription stops around the rho terminator (data not shown), indicating that the transcripts are processed/terminated differently at the 3′end. In summary, these data demonstrate that both *agrA* and *agrB* downregulate the level of *dinQ* full-length transcript whereas *agrB* is particularly important for down regulation of *+44 dinQ* (*dinQ-b*).

### Deleting *agrB* results in UV sensitivity

**Figure 3:**
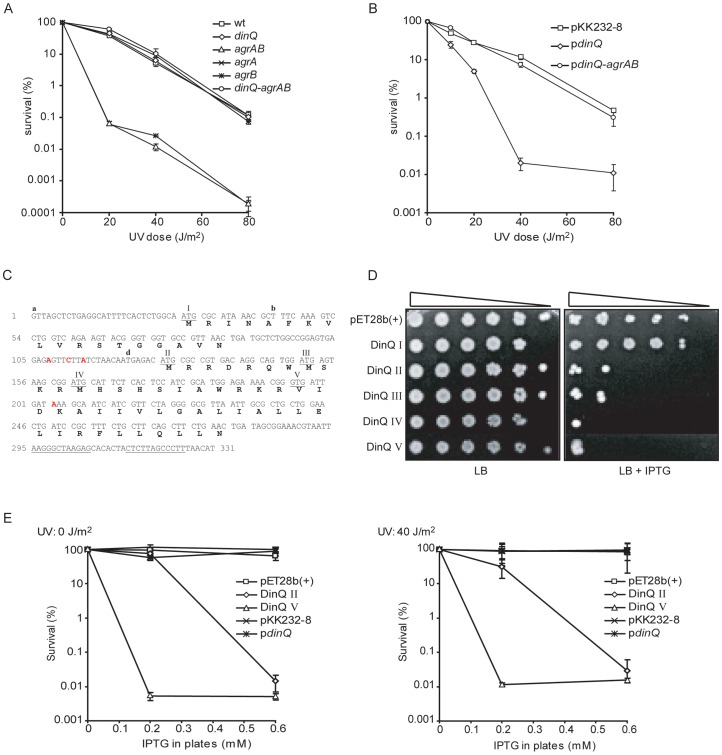
*DinQ*, *agrB*, and *agrA* phenotypes. (A) UV survival of *dinQ* (BK4040, diamond), *agrA* (BK4042, cross) and *agrB* (BK4043, star) single mutants, *agrAB* double mutant (BK4041, triangle) and the *agrAB dinQ* triple mutant (BK4044, circle) compared to wt (AB1157, square). The data are presented as mean of three independent experiments with standard deviation. (B) UV survival of wt (AB1157/pKK232-8, square), wt overexpressing *dinQ* (AB1157/pBK444, diamond) and wt overexpressing *dinQ-agrAB* (AB1157/pBK440, circle). The data are presented as mean of three independent experiments with standard deviation. (C) Sequence of the full-length *dinQ* transcript with annotated ORFs. The *dinQ* transcript contains two ORFs encoding putative peptides of 18 and 49 aa, in which the 49 aa ORF contain four putative start codons (underlined and assigned). DinQ II of 49 aa, DinQ III of 42 aa, DinQ IV of 38 aa and DinQ V of 27 aa which is translated from a GTG. Transcription terminator sequences are underlined. RNA start sites found in the primer extension experiments are assigned with **a**, **b** and **d**. Base mutations are assigned in red. (D) Serially diluted (10^−1^–10^−5^cells ml^−1^) log phase cultures of wt (ER2566) with expression vector pET28b(+) or vector constructs with putative *dinQ* encoded peptides (DinQ I–V) were spotted onto LB plates containing no IPTG (left panel) or 0.6 mM IPTG (right panel). Pictures were taken 1 day after incubation at 37°C. (E) Survival of wt (ER2566) overexpressing putative *dinQ* encoded peptides related to UV exposure. DinQ II (diamond), DinQ V (triangle), *dinQ* (asterisk) compared to vector controls pET28b(+) (square) and pKK232-8 (cross). Left panel shows survival of IPTG induced DinQ peptides without UV exposure and right panel shows survival of IPTG induced DinQ peptides when exposed to UV (40 J/m^2^).

The 31 nt antisense region in *agrB* gene (Figure 1C) indicate a function in antisense regulation of *dinQ* via RNA interference. This antisense sequence is partially complementary in *agrA* (Figure 1C) suggesting that both the *agrA* and *agrB* transcripts could base pair with the *dinQ* transcript. To ensure the function of *dinQ* when generating mutants, the deletions of *agrA* and *agrB* were made without destroying the *dinQ* promoter. *DinQ* belongs to the LexA regulon in *E. coli* [1] which regulates the SOS response. Several mutants of the SOS response, which play a direct role in DNA repair, display UV sensitivity. To examine the role of the *dinQ-agrAB* locus in the SOS response we tested the UV sensitivity of the various mutants (Figure 3A). The *agrB* single mutant and *agrAB* double mutant showed a significant increase in UV sensitivity compared to the isogenic wild type. In contrast, the *agrA* and *dinQ* single mutants and the *dinQ agrAB* triple mutant showed no UV sensitivity. Further we examined UV survival of the *agrB* mutant carrying a plasmid expressing the *agrB* gene (Figure S1), demonstrating that the mutant recovered completely. These data indicate a role for *agrB* in protection against UV exposure, in which the *agrB* transcript modifies the *dinQ* transcript. According to the northern analysis (Figure 2A) *agrB* represses accumulation of the *+44 dinQ/dinQ-b* transcript. It thus appears that the *+44 dinQ* product mediates the UV sensitivity of the *agrB* mutant.

### *AgrB* counteracts the UV sensitivity induced by *dinQ*

To further investigate the role of the *arsR-gor* intergenic region in UV protection, we cloned *agrA*, *agrB* and *dinQ* separately or the entire region containing both small RNAs and the *dinQ* gene into the cloning vector pKK232-8. Wild type cells transformed with pKK232-8-*dinQ* or pKK232-8 (control plasmid) showed the same viability during normal growth conditions (data not shown). In contrast, it was not possible to transform pKK232-8-*dinQ* into the *agrB* mutant (Table S2), indicating that the endogenous level of *agrB* in wild type cells is sufficient to inhibit *dinQ* expression from the pKK232-8-*dinQ* construct during normal growth. However, wild type transformed with pKK232-8-*dinQ* showed increased sensitivity to UV as compared to wild type transformed with the control plasmid (pKK232-8) (Figure 3B). When the wild type was transformed with constructs expressing *agrA* or *agrB* they did not increase sensitivity of the cells to UV (data not shown). Interestingly, wild type cells transformed with the plasmid carrying the entire *arsR-gor* locus, expressing all three genes, showed no UV sensitivity, demonstrating that *agrAB* can neutralize the UV sensitizing effect of *dinQ*. The *agrB* mutant (Figure 3A) and the wild type cells transformed with pKK232-8-*dinQ* (Figure 3B) displayed similar sensitivity to UV. These results suggest that the *agrB* transcript counteracts the UV sensitivity induced by *dinQ* expression.

### Slow growth of the *agrB* mutant

During construction of the *agrB* and *agrAB* mutants we observed that they form small colonies when plated on LB agar. To further investigate this growth phenotype we compared the growth rate in LB medium of the *agrB*, *agrA* and *dinQ* mutants and their isogenic wild type. OD_600_ was measured during growth and a sample was diluted and plated for viable counts. This experiment showed that only the *agrB* single mutant and *agrAB* double mutant grow more slowly than the wild type cells (Figure S2A). Also in glucose-CAA medium *agrB* mutant cells grew more slowly than wild type cells (Figure S2B and S2C). In another set of experiments we utilized flow cytometry to analyze whether DNA replication was affected in the growth impaired *agrB* mutant. We found that cells were smaller than normal with a reduced DNA concentration (Figure S2D). The total time for replication from origin to terminus was shorter in the mutant and the number of origins and replication forks per cell were fewer compared to the wild type. There was also a considerable heterogeneity in the observed reduction in cellular DNA concentration. This heterogeneity could be due to cell-to-cell differences in expression of the DinQ peptide.

### DinQ is translated from an alternative GTG start codon

The mechanism underlying the UV sensitive phenotype of *dinQ* in a multicopy situation or under constitutive upregulation in an *agrB* mutant is not clear. The *dinQ* gene contains two putative ORFs in which the second ORF contains three putative start codons (Figure 3C). In this work the corresponding peptides are termed DinQ I-IV. None of the putative DinQ peptides show homology to known peptides. To examine if any of the ORFs mediate the UV sensitivity shown by *dinQ*, each of the putative DinQ peptides (I–IV) were cloned into the expression vector pET28b(+) and expressed under control of IPTG. DinQ I displayed no increased sensitivity in absence or presence of UV, suggesting that the putative peptide translated from ORF I (Figure 3C) does not induce DinQ toxicity (Figure 3D and data not shown). In contrast, we observed that DinQ peptides II, III and IV showed a strong toxic/growth inhibitory effect even in absence of UV, demonstrating that the C-terminal amino acid sequence translated from start codon IV of the second ORF is sufficient to induce DinQ toxicity (Figure 3D). Next, expression of DinQ II was titrated with increasing concentrations of IPTG in absence or presence of UV (Figure 3E), showing that DinQ is highly toxic to the cells at very low doses of IPTG induction and the toxicity was UV independent.

**Figure 4:**
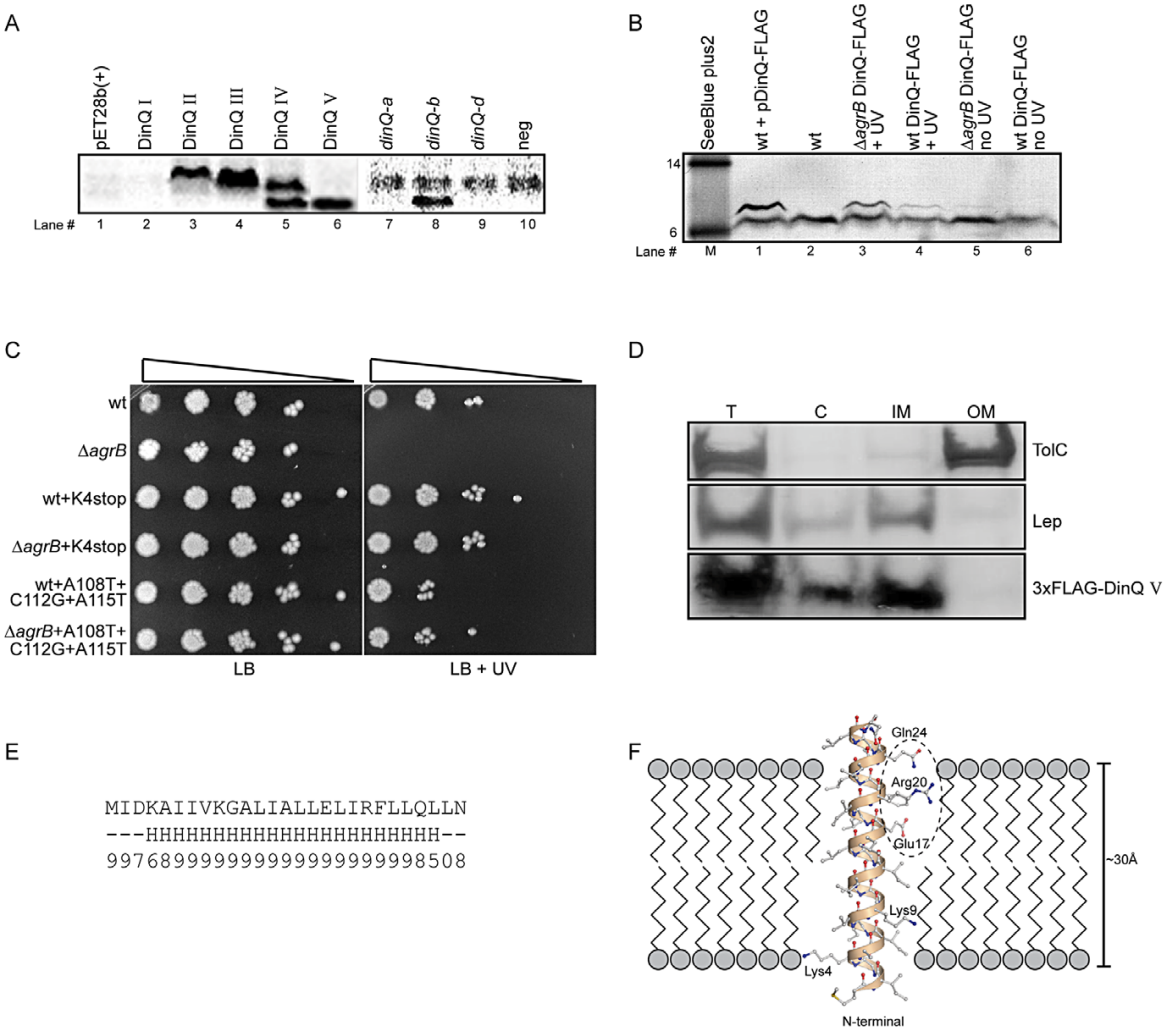
DinQ translation and localization. (A) Translation of putative DinQ peptides I–V expressed from pET28b(+) plasmid constructs (lanes 1–6) and PCR products corresponding to *dinQ-a, -b and -d* mRNAs (lane 7–10) were analyzed with coupled *in vitro* transcription/translation kits from Promega. The *E. coli* T7 S30 extract system for circular DNA was used to analyze DinQ expression from plasmid constructs while S30 extract system for linear templates was used to analyze PCR products (Promega). Labelling was carried out with [^14^C]-Leucine. (B) Western analysis of FLAG-tagged endogenous DinQ expression. Protein extracts from UV exposed (20 J/m^2^) control cells (BK4044) mixed with plasmid pCR2.1-DinQ-3×-FLAG in lane#1 (pos ctrl), wt (BK5300) in lane#2 (neg ctrl), UV exposed (50 J/m^2^) *ΔagrB* DinQ-3×FLAG (BK5372) in lane#3, UV exposed (50 J/m^2^) wt DinQ-3×FLAG (BK5370) in lane#4, unexposed *ΔagrB* DinQ-3×FLAG (BK5372) in lane#5, unexposed wt DinQ-3×FLAG (BK5370) in lane#6 was resolved by SDS-PAGE and analyzed by western blotting using Monoclonal ANTI-FLAG M2-Alkaline Phosphatase antibody (SIGMA). Gel migration was monitored relative to SeeBlue Plus2 prestained standard (Invitrogen) in kDa. Detection was carried out by NBT/BCIP color development substrates. The intensities of the DinQ bands was analyzed in three independent western blots with the program ImageJ (Rasband,W.S. and ImageJ, U. S. National Institutes of Health, Bethesda, MD, USA) and normalized against a cross reacting higher molecular weight protein band (not shown in Figure 4B). A cross reacting low molecular protein band of unknown origin is present in all lanes. (C) Serially diluted (10^−1^–10^−5^cells ml^−1^) log phase cultures of wt (BK5300), *ΔagrB* (BK5342), wt *dinQ-*K4stop (BK5350), *ΔagrB dinQ*-K4stop (BK5352), wt *dinQ*-A108T-C112G-A115T (BK5360) and *ΔagrB dinQ*-A108T-C112G-A115T (BK5362) were spotted onto LB plates and exposed to UV (0 J/m^2^ and 20 J/m^2^). Pictures were taken one day after incubation at 37°C. (D) Subcellular localization of DinQ in wt (ER2566) cells carrying plasmid pET28b(+)-3×FLAG-DinQ V. Subcellular fractions was resolved by SDS-PAGE and analyzed by western blotting using antibodies against TolC, Lep and FLAG. Lep and TolC detected inner- and outer membrane proteins, respectively. T: total protein; C: cytoplasmic fraction; IM: inner membrane fraction; OM: outer membrane fraction. (E) DinQ amino acid sequence with predicted secondary structure elements (H = helix, ‘-’ = other) and corresponding reliability index (range 0–9). (F) 3D modelling of DinQ as a regular α-helix embedded in a lipid membrane. Polar patch formed by residues Glu17, Arg20 and Gln24 encircled by dashed ellipse.

In another set of experiments we used a coupled *in vitro* transcription/translation *E. coli* T7 S30 extract to examine translation from pET28b(+) constructs encoding the putative peptides predicted from DinQ I–IV. Expression of DinQ I could not be detected whereas DinQ II–IV were highly expressed (Figure 4A, lanes 2–5). Notably, extracts with the DinQ IV construct produced two peptides of approximately 7.0 and 5.0 kDa, in which the smallest peptide indicates a fifth start codon. A closer inspection of the *dinQ* sequence uncovered a Shine Dalgarno motif within the DinQ IV sequence in optimal position to initiate translation at a GTG (termed codon V in Figure 3C), which encodes a putative peptide of 27 aa, termed DinQ V. To examine if the DinQ V peptide induces toxicity we cloned the sequence into pET28b(+), transformed the construct into wild type cells and monitored cell survival under the control of IPTG induction in presence or absence of UV exposure. DinQ V expression induced cell killing independently of UV treatment, suggesting that the sequence of peptide V is sufficient to mediate DinQ toxicity (Figure 3C and 3D). *In vitro* transcription/translation assays with the pET28b(+) DinQ V construct produced a peptide corresponding to the predicted molecular weight of peptide V (Figure 4A, lane 6).

Northern analysis identified five *dinQ* transcripts (Figure 2A; *dinQ a–e*), in which the start site were determined for transcript *a*, *b* and *d*. To assess the translational activity of the *in vivo dinQ* transcripts *a*, *b* and *d* we synthesized the corresponding PCR products carrying the T7 RNA polymerase promoter and added *E. coli* T7 S30 extract. Only the *dinQ-b/+44* transcript was translationally active, generating a peptide with a molecular weight similar to DinQ V, whereas the other transcripts were translationally inert (Figure 4A, lanes 7–10). Thus, it appears *in vivo* that the biologically active DinQ peptide (peptide V) is translated from the post transcriptionally modified *+44 dinQ* RNA.

To examine endogenous expression of DinQ *in vivo*, a 3×FLAG tag was inserted chromosomally in frame with the C-terminal of the *dinQ* gene in the wild type and *agrB* mutant. Western analysis revealed a faint band for the FLAG tagged DinQ peptide in the *agrB* mutant while the peptide was barely detectable in wild type (Figure 4B, lanes 5 and 6). In UV treated cells the DinQ level was about two fold higher in the *agrB* mutant as compared to wild type (Figure 4B, lanes 3 and 4). It thus appears that the phenotypes of the *agrB* mutant are not due to polar effects of extensive overexpression of DinQ.

Further, we introduced a chromosomal stop codon in the Lys4 position of *dinQ* in the *agrB* mutant and wild type (Figure 3C, base labeled in red). Survival experiments showed that UV resistance was restored to wild type level in the *agrB* mutant (Figure 4C), indicating that the UV sensitivity of the *agrB* mutant is caused by translation of a functional DinQ peptide. Next, we introduced three chromosomal point mutations in the *dinQ* up-stream sequence predicted to be involved in base pairing with *agrB* (Figure 3C, bases labeled in red). Exposure of these strains to UV in the *agrB* mutant background showed wild type levels of survival (Figure 4C). All together these results suggest that DinQ is translated into a peptide of 27 aa and the *agrB-dinQ* RNA interference is important for correct regulation of DinQ translation.

### DinQ is localized to the inner membrane

To determine the intracellular localization of DinQ, western analysis was performed on extracts after subcellular fractionation. As antibodies against the native DinQ peptides could not be obtained, we introduced a 3×FLAG epitope at the N-terminal of DinQ (peptide V). Spot assays on LB agar containing IPTG showed that the FLAG tagged peptide induced the same toxicity as the native peptide, demonstrating that the N-terminal FLAG tag had no effect on DinQ toxicity (data not shown). Cells were harvested at several time points after IPTG induction to test the level of expression by western analysis of whole cell extracts. The FLAG-DinQ peptide could not be detected before induction, but showed strong signals 5 to 40 min after induction (data not shown). To examine subcellular fractionation, antibodies against Lep and TolC were used as positive markers for inner and outer membrane fractions, respectively. The western blot showed that the inner and outer membranes are completely separated whereas the cytoplasmic fraction contains some contamination from the inner membrane (Figure 4D, middle panel). DinQ localized to the inner membrane but could not be detected in the outer membrane (Figure 4D, lower panel). The faint signal of the FLAG epitope in the cytoplasmic fraction is possibly due to cross contamination from the inner membrane. These data suggest that DinQ localization is confined to the inner membrane of *E. coli*.

### Computer modelling of DinQ predicts a single transmembrane peptide

Analysis of the DinQ amino acid sequence using the consensus secondary structure prediction tool Jpred3 [11] revealed that DinQ has high propensity to form a single α-helix. All residues except a few on each flanking terminal are predicted with high confidence to belong to the predicted α-helix (Figure 4E). With 20–22 residues in a single α-helix, the DinQ peptide could straightforwardly form a transmembrane helix of 6 full turns spanning more than 30 Å, as shown by modelling of DinQ using a regular α-helical template (Figure 4F). The two positively charged lysine residues (Lys4 and Lys9) are close to the phospholipid head groups, while particularly the charged Glu17, but also Arg20 and Gln24 may form a polar patch that can interact with other membrane embedded proteins (Figure 4F). The predicted single transmembrane peptide supports the localization of DinQ in the inner membrane (Figure 4D).

### DinQ is not affecting induction of the SOS response, filamentation, or mutagenesis

Previously, we showed that overexpressing another SOS inducible peptide, TisB, which encodes a small toxic inner membrane peptide, inhibits several SOS functions in wild type *E. coli* [12]. To determine whether DinQ affected induction of the SOS response we measured the level of *recA* and *lexA* mRNA in a mutant which constitutively overexpressed *dinQ*, *agrB* mutant or a *dinQ* deletion mutant (Table S1). Exponentially growing cells were exposed to UV and the amount of *recA* and *lexA* mRNA were determined prior to, and 20 min after irradiation by RT-qPCR. The expression levels of *recA* and *lexA* were similar in both mutants and wild type indicating that DinQ in contrast to TisB is not affecting regulation of the SOS response (Figure S3).

To investigate a potential role of *dinQ* in filamentation, we stained cell samples with acridine orange prior to and after UV exposure. All strains showed the same filamentation pattern, in which cells displayed short filaments 1 h after irradiation and long filaments after 2.5 h, indicating that DinQ is not involved in the filamentation process of *E. coli* (Figure S4). Next, we measured spontaneous and UV induced mutagenesis as the frequency of rifampicin resistant colonies in wild type and the *dinQ* and *agrB* single mutants. The results showed no significant differences in mutation frequency in the mutant strains as compared to wild type suggesting that DinQ is not altering spontaneous and SOS induced mutagenesis (data not shown).

### DinQ overexpression depolarizes the cell membrane

**Figure 5:**
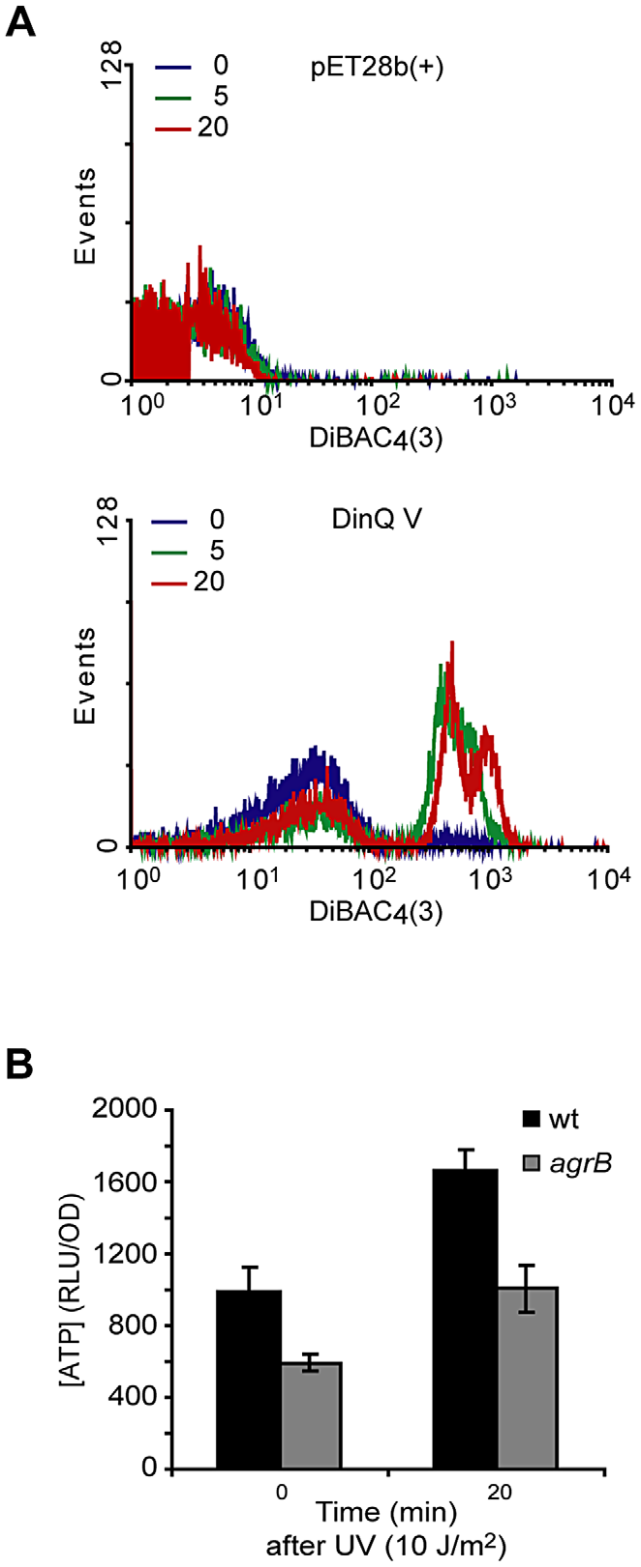
Effects of DinQ on membrane potential and intracellular ATP. (A) Exponentially growing cells were exposed to IPTG for 0, 5 and 20 min followed by measurement of DiBAC_4_(3) fluorescence. An increase in fluorescence intensity is observed as a consequence of membrane depolarization. Upper panel displays wt (ER2566) cells carrying expression vector pET28b(+). Lower panel displays wt (ER2566) cells overexpressing DinQ V (pET28b(+)-DinQ V). The graphs are representative of at least three repetitions. (B) The staple diagram shows *in vivo* levels of ATP before and 20 min after UV exposure of wt (AB1157) and mutant *agrB* (BK4043) cells. ATP concentrations were determined by a luciferase-based assay (Promega) and the activity is shown as RLU/OD (relative light units related to optical density). The data are presented as mean of three independent experiments with standard deviation.

To examine whether high levels of DinQ induce changes in membrane potential we tested the ability of *E. coli* cells overexpressing DinQ to take up the dye DiBAC_4_(3) [bis-(1,3-dibarbituric acid)-trimethine oxanol]. The quantity of dye entering cells is proportional to membrane polarization, the less polarised the membrane the greater the quantity entering the cells and so increased fluorescence intensity due to binding to the membrane and intracellular components [13]. Cells were analyzed 5 and 20 min after IPTG induction of DinQ expression, incubated with DiBAC_4_(3) for 20 min and analyzed by flow cytometry (Figure 5A). No changes were observed for the plasmid control pET28b(+). However, IPTG induction of DinQ (peptide V) showed a rapid increase in DiBAC_4_(3) uptake (Figure 5A), suggesting that elevated levels of DinQ depolarize the cell membrane. This data indicates that DinQ overexpression interferes with membrane polarity and could therefore lead to a loss of viability.

### DinQ overexpression decreases the intracellular ATP concentration

Subcellular fractionation of *E. coli* showed that DinQ is localized to the inner membrane (Figure 4D). DinQ is predicted to be a hydrophobic single transmembrane peptide that might compromise inner membrane integrity (Figure 4F). We speculated that if DinQ affected the proton motive force, it would affect ATP production and intracellular ATP concentration. The intracellular ATP concentration was measured in wild type cells and in the *agrB* mutant, using a quantitative luciferase-based assay. This experiment showed that the concentration of ATP in the *agrB* mutant was about 50% of the concentration measured in wild type cells (Figure 5B). Further, UV exposure increased the ATP concentration 0.6 fold in both cell types. Thus, it appears that insertion of the DinQ peptide into the inner membrane of *E. coli* impairs the energy supply in the form of ATP.

### DinQ modulates conjugal recombination

**Figure 6:**
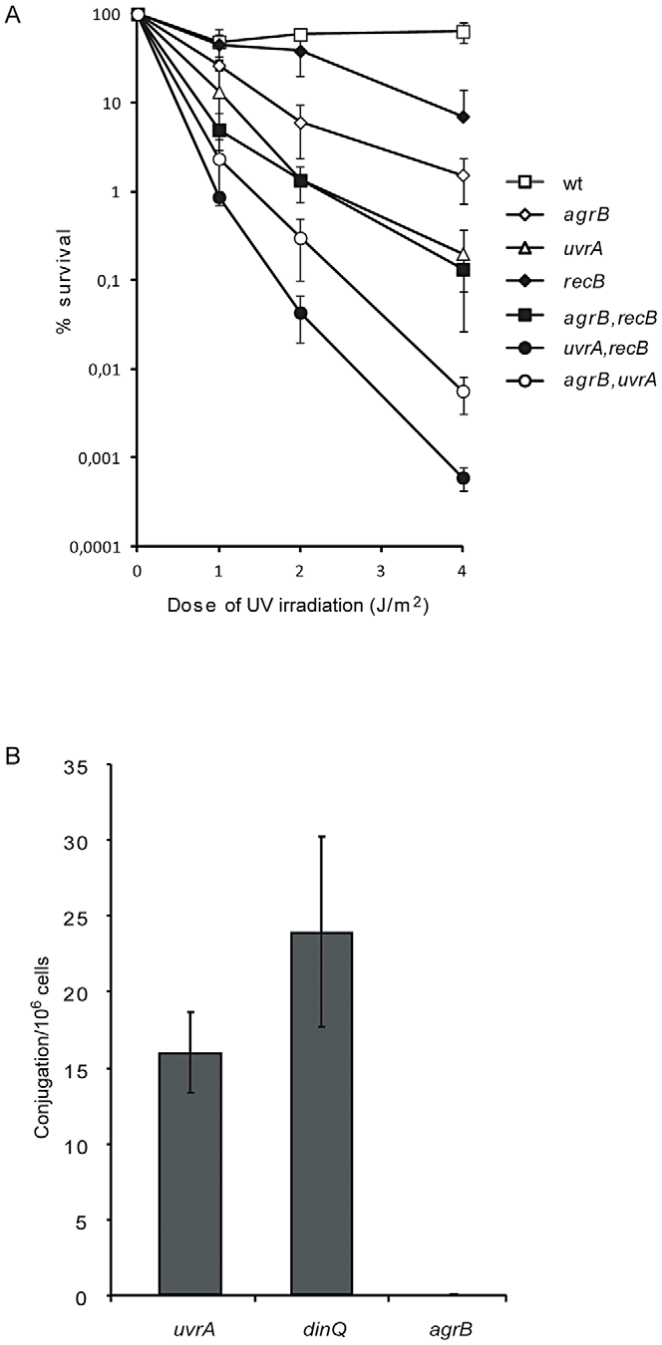
Genetic interactions between *agrB* and *uvrA*/*recB* and recombination frequency of *agrB*. (A) Dilutions of exponentionally growing wild type (AB1157, open square), *agrB* (BK4148, open diamond), *uvrA* (BK4180, open triangle), *recB* (BK4110, black diamond), *agrB recB* (BK4112, black square), *uvrA recB* (BK4183, black circle) and *agrB uvrA* (BK4182, open circle) cells were plated on LB plates and exposed to UV irradiation. Colonies were counted one day after incubation at 37°C. The data are presented as mean of three independent experiments with standard deviation. (B) Recombination frequency of *uvrA* (BK4180), *dinQ* (BK4040) and *agrB* (BK4043/BK4148) determined by Hfr conjugation assays. The data are presented as mean of three independent experiments with standard deviation.

The *agrB* mutant displayed sensitivity to UV suggesting that DinQ could have a role in the repair of UV induced DNA damage. Both nucleotide excision repair (NER) and recombinational repair (RR) counteract the genotoxic effects of UV irradiation. NER is required for the repair of UV induced photoproducts such as thymine dimers and cyclopyrimidine dimers, while RR is required for the repair of strand gaps and double strand breaks. To examine if DinQ is involved in NER we analysed UV sensitivity of the *uvrA agrB* and *uvrA dinQ* double mutants as compared to the single mutants. The survival analysis showed an additive effect between *agrB* and *uvrA* (Figure 6A), whereas *uvrA* and *dinQ* showed no additional effect (Figure S5). These data indicate that elevated levels of DinQ in the *agrB* mutant sensitize the cell to UV via a pathway which is independent of NER. To examine the role of DinQ in recombination, mutant strains *dinQ*, *agrB*, *recB*, (*recB agrB*) and (*dinQ recB*) were exposed to UV irradiation. The double mutant (*recB agrB*) was slightly more sensitive to UV than the *agrB* single mutant (Figure 6A), whereas *recB* and *dinQ* showed no additional effect (Figure S5).

To further examine the role of DinQ in recombination we performed Hfr conjugation assays with a donor strain containing *Tn10*, which carries the tetracycline resistance gene integrated in its chromosome and *agrB*, *dinQ* and *uvrA* single mutants as recipient strains. We used the *uvrA* mutant as control strain since UvrA is not involved in recombination (and carry the kanamycin resistance gene required to detect the recipient). Hfr conjugation of *Tn10* was at least 400-fold more efficient in *dinQ* and *uvrA* mutants as compared to *agrB* (Figure 6B), suggesting that elevated levels of DinQ inhibit recombination. To examine if the conjugational process itself is affected in an *agrB* recipient we performed plasmid conjugation assays with a donor strain carrying an F′-plasmid with tetracycline resistance and the same recipient strains as in the Hfr conjugation experiment. Hfr conjugation differs from F′-plasmid conjugation in that transfer of genes after Hfr requires recombination whereas the F′-plasmid does not recombine in the recipient. In these experiments we find no differences in plasmid conjugation frequencies between the *dinQ*, *uvrA* and *agrB* recipient strains (data not shown). In sum, these results suggest that recombination is inhibited in the *agrB* mutant during Hfr conjugation, but not in the transfer and uptake of DNA or survival of the *agrB* recipient. It thus appears that elevated levels of DinQ affect the recombination process.

### Extended duration of nucleoid compaction in *agrB* mutant cells after UV irradiation

**Figure 7:**
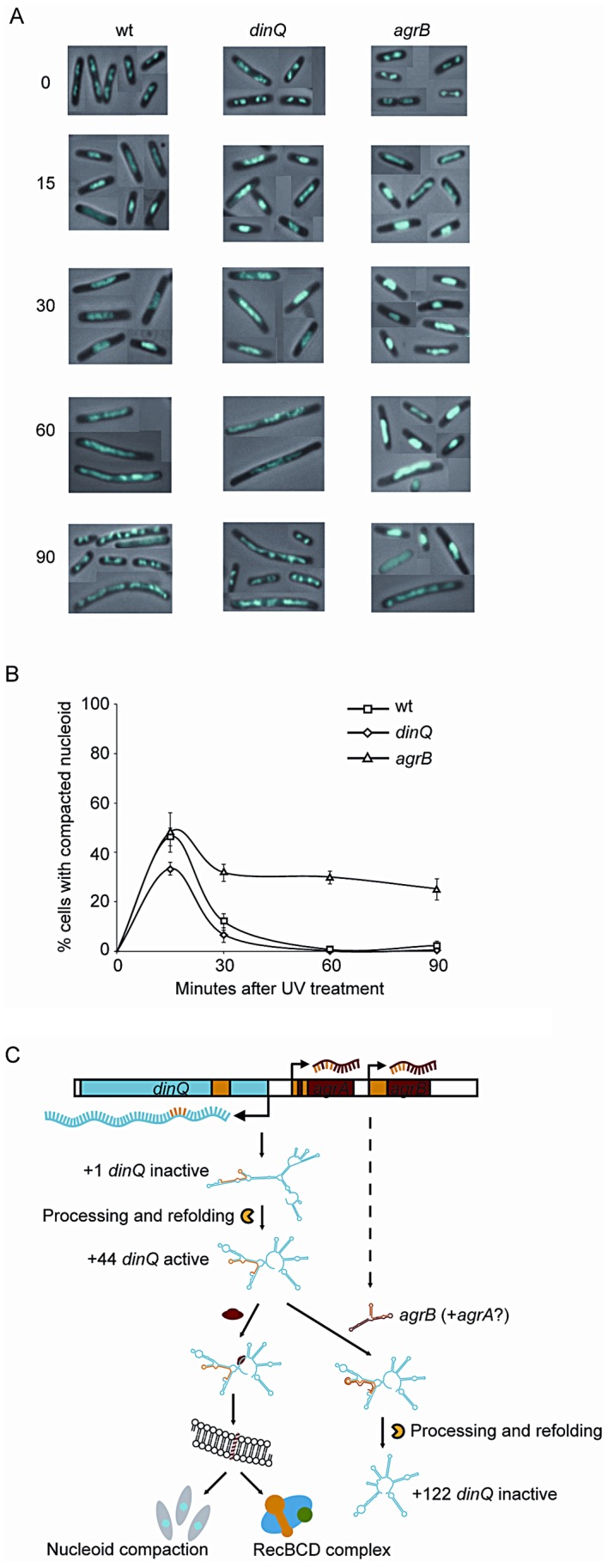
Nucleoid compaction during DNA repair and model of regulation and mechanism of action for DinQ. (A) Exponentially growing wt (AB1157), *dinQ* (BK4040) and *agrB* (BK4043) cells in glucose-CAA medium were exposed to UV (3 J/m^2^). Samples were harvested at the indicated time points and cells were fixed, stained with Hoechst 33258 and mounted on microscope slides. The panel of pictures shows a mosaic of representative cells. (B) The fraction of cells with compact nucleoids (i.e. cells containing one or two non-extended nucleoids instead of two or four normal nucleoids, respectively) were scored for each time point. The average of three experiments and a total of 250–500 cells were counted for each time point. Error bars indicate the standard deviation. Note that normal nucleoids as well as extended, diffuse nucleoids were scored as “non-compact”. (C) This model summarizes all the data presented in this work, including regulation of transcription and translation, and the proposed mechanism of action at the inner cell membrane. All RNA secondary structures are computer models generated by the mfold web server software.

In dividing cells, replication forks are stalled by DNA lesions that impair DNA unwinding or block synthesis by the DNA polymerase subunits. In *E. coli*, UV lesions cause a delay in DNA synthesis for a period of time while stalled forks undergo repair. Fluorescence microscopy of Hoechst stained cells has demonstrated that the DNA often forms a compact structure during this phase, and suggests that the nucleoids undergo a major reorganization after UV exposure [14]. To investigate whether DinQ affects nucleoid organization, we used this technique to examine the shape and size of the nucleoids at different time points after UV exposure. In undamaged cells the nucleoids have characteristic shapes and numbers depending on the growth medium. When grown in glucose-CAA medium most cells have two nucleoids and some (the largest cells) have four (Figure 7A, 0 min). Microscopy of cells 15 min after UV irradiation shows that all the wild type cells had lost the normal nucleoid morphology. In approximately 45% of the cells the nucleoids had been rearranged into a highly compact structure, whereas in the rest of the cells the nucleoids were found to be extended throughout the cells (Figure 7A and 7B). Sixty minutes after UV irradiation all cells were found to contain extended nucleoids. After 90 min approximately 30% of the cells had divided and contained nucleoids with normal morphology. In the *agrB* mutant the degree of nucleoid compaction was similar to that of wild type cells at 15 min after UV exposure (Figure 7A and 7B). However, in the period from 30 to 90 min 25–30% of the nucleoids of the *agrB* mutant cells were still locked in a compact state whereas a decreasing number of the wild type cells contained compact nucleoids. The result indicates that the transition from compact to extended nucleoid was inhibited in the *agrB* mutant.

We also investigated the *dinQ* mutant with respect to nucleoid morphology after UV irradiation. At the 15 min time point approximately 35% of *dinQ* mutant cells contained a compact nucleoid compared to about 45% of wild type cells (Figure 7B). This indicates that the compaction process might be affected in cells without DinQ. At 30, 60 and 90 min similar numbers of cells with compact and extended nucleoids were found in the *dinQ* mutant compared to in wild type cells (Figure 7B). The results indicate that cells lacking DinQ have an impaired ability to form a compact nucleoid structure after UV irradiation. Taken together the data reveals that the presence of DinQ is required in order to execute a transformation of nucleoid morphology in response to UV damage, and that overexpression of DinQ leads to a delay in decompaction and extension of the nucleoid during the later stages of the response.

In conclusion, *DinQ* is under the control of the SOS response and the *agrB* antisense RNA, and expresses a single transmembrane peptide that has an effect on nucleoid compaction and when overexpressed on conjugal recombination (summarized in Figure 7C).

## Discussion

Recently, a search for small proteins in *E. coli* could not detect any translation of the DinQ ORF [6], [7]. In this paper we characterize the *arsR-gor* intergenic region of *E. coli*, which contain the SOS inducible *dinQ* gene and two constitutively expressed small RNAs, *agrA* and *agrB*, with antisense complementarity to the *dinQ* gene. We show that DinQ is tightly regulated at both the transcriptional and translational level. Five different *dinQ* transcripts were identified in which only the endonucleolytic +44 transcript (*dinQ-b*) is translationally active. Further, *agrB* appears to repress accumulation of *dinQ-b* by RNA interference. Unexpectedly, DinQ is not translated from any of the three ATG codons within the ORF but from an alternative GTG start codon, encoding a 27 aa peptide which is localized in the inner membrane. The *agrB* mutant, which expresses elevated levels of *dinQ-b*, displays increased sensitivity to UV induced DNA damage and an impaired frequency of conjugal recombination. It thus appears that DinQ could be involved in the modulation of homologous recombination. Small single transmembrane peptides such as DinQ may be key regulators of processes at the inner membrane, in which their expression are strictly regulated to avoid toxicity. In summary, the experiments presented in this paper provide insights into the complex regulation of *dinQ* and suggest a mode of action within the bacterial inner membrane (Summarized in Figure 7C).

### Regulation and processing of *dinQ* RNA

Previous attempts to identify a translation product for *dinQ* have been unsuccessful, presumably because transcription and translation of *dinQ* are strictly regulated. First, the promoter region of the *dinQ* gene contains two LexA operators with different HI, which may suggest differential expression of the transcript early and late in the SOS response. Second, we identified two novel small non coding RNAs *agrA* and *agrB* with sequence complementarity to *dinQ* in the *arsR-gor* region that regulate *dinQ* by RNA interference. Notably, only the *agrB* RNA repressed the translational active +44 *dinQ* transcript (*dinQ-b*) whereas both sRNA repressed the primary translationally inactive *dinQ* transcript. Further, only the *agrB* antisense RNA counteracts DinQ toxicity. As such the *dinQ-agrAB* system appears to conform to the definition of a classical type I toxin-antitoxin (TA) system. It thus appears that the *dinQ*/*agrB* complex inhibit the endonucleolytic cleavage producing the active mRNA (*dinQ-b*). Presumably, these antisense sRNAs have been tandemly duplicated in the genome, in which *agrA* has partly degenerated and appears to be non-functional due to less antisense sequence complementarity with the *dinQ* sequence as compared to *agrB*.

The genomic organization and mode of antisense regulation of *dinQ* in the *arsR-gor* region resembles regulation of another SOS induced TA system, *tisAB* [15], [16]. Similar to the *dinQ* RNA, endonucleolytic processing of the primary *tisAB* transcript is required to generate an active mRNA producing the toxic TisB peptide. Further, the *tisAB* locus contains an antisense RNA, IstR-1 that inactivates the translationally active mRNA by RNaseIII dependent cleavage. It appears that *agrB* may have a similar role in RNase dependent cleavage of the translationally active *dinQ* mRNA (*dinQ-b*). In addition, Darfeuille *et al* revealed that the antisense RNA IstR-1 inhibits translation of the TisB toxin by competing with standby ribosomes binding upstream of the translation initiation region (TIR). It is proposed that binding to the “standby” site is required for initiation of protein synthesis at the highly structured *tisB* TIR by ribosome sliding to the transiently open TIR [16]. In a similar manner, we speculate that the *agrB* antisense RNA regulates/inhibits translation from the active +44 transcript by binding a potential “standby” site upstream of the *dinQ* TIR.

### DinQ mode of action

In order to investigate DinQ biochemically, we have attempted to purify DinQ, including fusion peptides. However, all attempts to purify DinQ as well as chemical synthesis of the peptide failed because of the hydrophobic nature of the peptide. Further, a general feature of small hydrophobic peptides including DinQ is the lack of obvious phenotypes associated with their inactivation [17]–[20]. As an alternative strategy we characterized the phenotype of augmented DinQ in an *agrB* mutant. The DinQ concentration in the *agrB* mutant after UV treatment is elevated only two fold as compared to the wild type, indicating that the DinQ levels in the *agrB* mutant is physiologically relevant. Of particular interest was the 400-fold reduction in the recombination frequency in the *agrB* mutant as compared to wild type cells, suggesting that augmented DinQ inhibits recombination in the *agrB* mutant. However, genetic data suggests that DinQ may also play a role in UV protective mechanisms independent of recombination.

It appears that the large, ordered hyperstructures involved in homologous recombination are associated with the cell membrane [21]. The hyperstructures are dynamic and their size is dependent on the extent of the initial or ongoing DNA damage. The DinQ peptide is localized in the inner membrane of the cell and it is tempting to speculate about a role for DinQ in regulating DNA repair hyperstructures at the inner membrane. In addition, the prolonged period of nucleoid condensation in the *agrB* mutant may contribute to the impairment of DNA repair processes. Although such a direct role for DinQ is speculative, several small hydrophobic peptides have been demonstrated to modulate membrane dependent processes. The B1500 protein (65 aa) interacts with the PhoQ sensor [18], the 30 aa protein MgtR (30 aa) interacts with MgtC [19], the KdpF protein (29 aa) is part of the Kdp complex [9] and the SidA protein (29 aa) interacts directly with FtsW and FtsN [22].

The intracellular concentration of ATP was reduced in the *agrB* mutant compared to the wild type both before and after UV exposure. In wild type cells UV irradiation induces a two fold increase in ATP concentration during the first 20 to 30 min after exposure, and the increase is RecBC dependent [23]. These data suggest that loss of *agrB* and thereby excess of DinQ limit the cellular energy supply and may also explain some of the observed phenotypes. Our FLAG tag experiments revealed that DinQ increases only two fold in an *agrB* mutant and this apparently modest increase is sufficient to mediate dramatic effects on conjugal recombination rates, membrane depolarization, ATP levels and nucleoid reorganization. In a wild type cell population the level of DinQ translation is kept strictly under control by the LexA repressor, antisense *agrB* RNA and *dinQ* RNA processing, so for the majority of cells DinQ may never reach a level high enough to mediate the effects observed in an *agrB* mutant. Heterogeneity in the expression of LexA repressed genes has been observed by studying SOS promoter fusions in combination with imaging techniques and a subpopulation of cells clearly have a stronger SOS induction [24]–[26]. It is tempting to speculate that a higher level of DinQ is reached only in a subpopulation of cells where SOS induction is particularly strong or long lasting leading to a permanent or temporary *agrB* phenotype. Such an effect has been proposed for some toxin/antitoxin pairs in promoting formation of persister cells [27], [28]. To gain a more detailed knowledge about the biological function of DinQ the *agrB* mutant could be an excellent model for studying the effects of DinQ and similar hydrophobic peptides in bacterial subpopulations.

## Materials and Methods

### Strains, plasmids, and media

The experiments were carried out in an AB1157 background [29]. Except for chromosomal point mutations and chromosomal 3×FLAG tags all mutants were made in strain BW25113-pKD46 [30] and introduced into AB1157 via T4GT7 transduction [31]. The *agrA* (BK4042), *agrB* (BK4043) and *dinQ* (BK4040) single mutants were made by deleting each of the genes and introducing a *kan^r^* cassette. Next, the *agrAB* double mutant (BK4041) was generated by deleting both genes and introducing a *kan^r^* cassette. To construct a triple mutant the entire *arsR-gor* intergenic region containing *dinQ*, *agrA* and *agrB* was deleted (BK4044) and replaced with the *kan^r^* cassette. Table S1 summarizes all strains used and generated in this work. Vector pKK232-8 (10–25 copies pr cell in *E. coli*) contains a promoter less *cat* gene allowing selection of DNA fragments containing promoter activity [32]. pBK440 (dinQ-agrAB)/pBK444 (dinQ) is based on the vector pKK232-8 (Pharmacia) with a 2065/415 bp insert respectively from the intergenic region between *arsR*-*gor* in MCS, resulting in a plasmid that expresses *E. coli dinQ* from its own SOS inducible promoter. Cloning primers are listed in Table S3. Expression plasmids pET28b(+)-DinQ I, pET28b(+)-DinQ II, pET28b(+)-DinQ III, pET28b(+)-DinQ IV and pET28b(+)-DinQ V contain the DinQ I to V ORFs inserted in the *NcoI*-*BamHI* restriction sites of the pET28b(+) vector (Novagen). Chromosomal point mutations in *dinQ* to either introduce a premature translational stop codon in DinQ ORFV (K4stop) or introduce three point mutations in the *agrB* antisense region of *dinQ* (A108T, C112G, A115G) or to introduce a chromosomal DinQ C-terminal 3×FLAG tag were made by splicing PCR products with overlap extension (SOEing PCR) and recombine the final SOEing PCR product into a MG1655 background as described [30]. All SOEing products contained a flanking *kan^r^* cassette close to the *arsR* gene to facilitate selection of recombinants. To avoid unwanted recombination between the *kan^r^* cassette and the point mutations or the 3×FLAG tag during strain construction the SOEing products were transformed into strain BK5444-pKD46 which lacks the chromosomal *dinQ-agrAB* locus and where insertion/recombination of the SOEing products is possible only in the flanking homologous DNA sequences. The final PCR products were transformed into MG1655 containing pKD46. Cells were cured for pKD46 and insertions verified by PCR and sequencing. Details of strain construction and oligos used are listed in Tables S1 and S3, respectively. GenScript Corp. gene service constructed DinQ II and V with an N-terminal 3×FLAG tag that was inserted in the *NcoI*-*BamHI* restriction sites of the pET28b(+) vector (Novagen). Cells were grown in LB- or K-medium [33] with appropriate antibiotics (100 µg/ml ampicillin and 50 µg/ml kanamycin). For the nucleoid compaction studies cells were grown in AB minimal medium [34] supplemented with 1 µg/ml thiamine, 0.2% glucose and 0.5% casamino acids.

### *In vitro* transcription/translation

*In vitro* transcription/translation on circular pET28b(+) templates or linear PCR products were performed according to Promegas protocols *E. coli* T7 S30 Extract System for Circular DNA and *E. coli* S30 Extract System for Linear Templates, respectively, with [^14^C]-Leucine as radiolabeled amino acid. The translation products were analysed by SDS-PAGE and visualized on Typhoon 9410 (Amersham).

### Protein fractionation and membrane localization

Aliquots of exponentially growing ER2566/pET28b(+)-DinQ V were harvested by centrifugation 20 min after IPTG induction (1 mM). Cells were resuspended in 4 ml 50 mM phosphate buffer pH 7.2 and sonicated three times for 15 sec. Further fractionation was performed as described by [20]. Proteins from all fractions were acetone precipitated 1∶1 overnight at -20°C, pellets after centrifugation was resuspended in 4× NuPAGE sample loading buffer (Invitrogen) and loaded onto 10% NuPAGE Novex Bis-Tris gels (Invitrogen).

### Flow cytometry

Cells were grown to OD_600_≈0.4 in LB and induced with IPTG (1 mM). At 0, 5 and 20 min culture samples were diluted 1∶10 in filtered AB minimal medium [34] +10 µg/ml DiBAC_4_(3) (Sigma-Aldrich). After 20 min incubation in the dark at room temperature, cells were analysed in a Flowcytometry LSRII (Becton Dickinson) equipped with an argon ion laser and a krypton laser (both Spectra Physics). DiBAC_4_(3) was detected using 488 nm laser. The distribution of DiBAC_4_(3) fluorescence was plotted on a logarithmic scale. The data obtained was analyzed by winMDI software.

### ATP assay

Cell aliquots were harvested before and 20 min after induction with IPTG (1 mM) and washed once in 50 mM Tris-acetate pH 7.75. ATP was extracted from washed cells by 1% trichloroacetic acid (TCA) in 50 mM Tris-acetate pH 7.75 for 10 min. Tris-acetate pH 7.75 was added 1∶10 to obtain optimal pH of 7.75 before mixing with rL/L reagent (ENLITEN ATP assay, Promega) at room temperature. The amount of ATP extracted (RLU value) was measured with 20/20 Luminometer (Turner Designs) and related to the OD_600_ for each sample.

### Frequency of recombination by Hfr conjugation

Aliquots of exponentially growing recipient strains *dinQ* (BK4040), *agrB* (BK4043) and *uvrA* (BK4180) were mixed in equal volumes with donor strains BW7623 (with the tetracycline resistance gene, Tn10, integrated in its chromosome) or ER2738 (carrying a tetracycline resistance conjugative plasmid) and incubated at 37°C for 30 minutes. BW7623 was used to examine chromosomal transfer to the recipient strains (recombination dependent) whereas ER2738 was used for plasmid conjugation. Cells were vortexed thoroughly and spread on selective LB plates. Hfr recombination rate and plasmid conjugation rate was calculated as number of recombinants/conjugated cells pr 10^6^ cells.

### Nucleoid compaction

Exponentially growing wt (AB1157), *dinQ* (BK4040) and *agrB* (BK4043) cells were UV irradiated with 3 J/m^2^ while stirring. 1.5 ml samples were taken at 0, 15, 30, 60 and 90 min after irradiation. Washed once and resuspended in 100 µl cold, filtered TE buffer. Then 1 ml of cold, filtered 77% ethanol was added for fixation. Fixed cells were mounted on a poly-L-lysine coated microscope slide and the DNA was stained with Hoechst 33258 (5 µg/ml, Sigma) in mounting medium (40% glycerol in PBS pH 7.5). Visualization of stained cells was performed using a Leica DM6000B phase-contrast/fluorescence microscope equipped with a 63× objective and a BP340-380 excitation filter. Pictures were taken using a Leica DFC350 FX digital camera that was connected to a computerized image analysis system (LAS AF software, version 2.0.0, Leica). The fluorescent image was merged with the phase-contrast image.

## Supporting Information

Figure S1 Complementation. Serially diluted (10^−1^–10^−5^cells ml^−1^) log phase cultures of wt (AB1157) or *agrB* (BK4043) with expression vector pKK232-8 or vector constructs with *agrB* (pKK232-8-*agrB* = pBK446) were spotted onto LB plates and exposed to 0 J/m^2^ UV (left panel) or 50 J/m^2^ UV (right panel). Pictures were taken 1 day after incubation at 37°C.
(TIF)

Figure S2 Growth curves, DNA histograms, and cell cycle analysis of wild-type cells and *agrB* mutant cells. (A) Growth rates of wt (AB1157, diamond) compared to *dinQ* (BK4040, square), *agrAB* (BK4041, triangle), *agrA* (BK4042, cross), *agrB* (BK4043, star) and *dinQ-agrAB* (BK4044, open circle). (B, C and D) Cell cycle analysis and flow cytometry of wt (AB1157) (B) and *agrB* (BK4043) (C). Cells were grown exponentially in AB minimal medium supplemented with 1 µg/ml thiamine, 0.2% glucose and 0.5% casamino acids at 37°C to OD_450_ = 0.15. Cells were either harvested or treated with 300 µg/ml rifampicin and 10 µg/ml cephalexin for 5 generations. The left and middle panels show DNA histograms of exponentially growing cells and cells treated with rifampicin (rif) and cephalexin (cpx), respectively, where the number of cells is plotted against the number of chromosome equivalents. The number of chromosome equivalents for rifampicin- and cephalexin-treated cells corresponds to the number of origins at the time of drug action. The right panel shows a schematic diagram of the cell cycle. The timeline at the bottom shows the generation time (τ) as well as the times for initiation (ai) and termination (at) of replication. Each horizontal bar represents one generation where the “current” generation is denoted k, the “mother” generation k-1 and the “grandmother” generation k-2. The C period (elongation) is colored red and the D period (segregation and cell division) is colored grey as one replication cycle is followed through three (AB1157, B) and two (BK4043, C) generations. Cells showing the replication pattern at the time of initiation and termination of replication are drawn on top of the diagram. A black dot and a grey circle represent the origin and the chromosome, respectively. The C+D period was determined from the initiation age, generation time and number of generations spanned by C+D. The initiation age and number of generations spanned were found from the rif/cpx histogram and the relative lengths of C and D were found from the exponential histogram. Relative DNA content per mass of the *agrB* mutant as compared to wt is calculated and presented in the table (D). For details on cell cycle analysis and flow cytometry, see [14]. From the cell cycle analysis we found that the wt (AB1157) cells (B) initiated replication at four origins in the “grandmother” generation. The C period was determined to be 55 minutes while the D period was determined to last one generation (35 minutes). The *agrB* mutant (BK4043) cells (C) were found to initiate at two origins when newborn in the “mother” generation. Since the replication and segregation periods (C+D) spanned fewer generations, the C period was found to be shorter than in wt cells (41 minutes compared to 55 minutes). The D period was determined to last for one generation (41 minutes) as in wt cells. The values found for the cell cycle parameters are average values for the respective cell cultures. Especially the mutant cells have a large cell-to-cell variability as can be seen in the rif/cpx histogram which shows that some cells have initiated replication, but not divided, and some cells have divided but not initiated replication.
(TIF)

Figure S3 RT–qPCR showing stable *recA* and *lexA* mRNA concentrations with regards to *rrsB* in various *dinQ* regulon mutant strains both before and after UV exposure. The graphs show the mRNA concentrations of the SOS response regulators recA mRNA (A) and lexA mRNA (B) relative to the *rrsB* transcript before and after UV exposure. The relative concentrations in various mutant strains: *dinQ* (BK4040), *agrAB* (BK4041), *agrA* (BK4042), *agrB* (BK4043), *dinQ-agrAB* (BK4044) and the isogenic wt reference strain (AB1157) are compared. No significant difference was seen between the different strains indicating that the disrupted regions do not affect the induction of the SOS response at the transcription level. cDNA was synthesized from DNaseI treated total RNA (1 µg) using the High Capacity cDNA Reverse Transcription Kit (ABI) according to the manufactures' instructions. Power SYBR Green PCR MasterMix together with a StepOnePlus Real-time PCR System (ABI), cDNA (5 ng) and rrsB, recA or lexA primers (Table S2) to generate real-time plots that were automatically processed by the StepOne Software v2.0.1 to calculate cycle threshold (*C_t_*) values. The primers were automatically selected using the Primer Express 3.0 software (ABI). Four independent samples were run in quadruplet to generate a mean *C_t_* value relative to the rrsB (16S ribosomal RNA) transcript, Δ*C_t_*, which was used as the endogenous control. The Δ*C_t_* values fall after UV exposure due to the stable larger amount of rrsB transcript and a rising but reduced amount of recA or lexA transcript.
(TIF)

Figure S4 Filamentation. Fluorescence microscopy of UV exposed (50 J/m^2^) strains as indicated followed by growth for 1 and 2.5 h. Cells were grown in K-medium and stained with acridine orange. Each image is quantified and median values in µm are given together with number of cells measured (in parentheses).
(TIF)

Figure S5 Survival of (*dinQ,recB*) and (*dinQ, uvrA*) mutants. Serially diluted Serially diluted (10^−1^–10^−5^cells ml^−1^) log phase cultures of wt (AB1157), Δ*dinQ* (BK4140), *uvrA::kan* (BK4180), *ΔdinQ,uvrA* (BK4141), *recB::kan* (BK4110) or Δ*dinQ,recB::kan* (BK4142) were spotted onto LB plates and exposed to 4 J/m^2^ (right panel), unexposed panels to the left. Pictures were taken 1 day after incubation at 37°C.
(TIF)

Table S1 Characteristics of bacterial strains and plasmids used in this study.
(DOCX)

Table S2 Transformation efficiency. Exponentially growing cells of wt (AB1157) and *agrB* (BK4043/BK4180) were transformed by electroporation with plasmids pKK232-8 and pBK444 (*dinQ*). Dilutions were spread on LB plates and transformed cells were counted.
(DOCX)

Table S3 Oligonucleotides used in this study.
(DOCX)

## References

[1] Fernandez De Henestrosa AR, Ogi T, Aoyagi S, Chafin D, Hayes JJ, Ohmori H, et al (2000) Identification of additional genes belonging to the LexA regulon in Escherichia coli. Mol Microbiol 35: 1560–1572. 10760155 10.1046/j.1365-2958.2000.01826.x

[2] Courcelle J, Khodursky A, Peter B, Brown PO, Hanawalt PC (2001) Comparative gene expression profiles following UV exposure in wild-type and SOS-deficient Escherichia coli. Genetics 158: 41–64. 11333217 10.1093/genetics/158.1.41 PMC1461638

[3] Zuber P (2001) A peptide profile of the Bacillus subtilis genome. Peptides 22: 1555–1577 doi:DOI:10.1016/S0196-9781(01)00492-2. 11587785 10.1016/s0196-9781(01)00492-2

[4] Ibrahim M, Nicolas P, Bessieres P, Bolotin A, Monnet V, et al (2007) A genome-wide survey of short coding sequences in streptococci. Microbiology 153: 3631–3644. 17975071 10.1099/mic.0.2007/006205-0

[5] Alix E, Blanc-Potard ABa (2009) Hydrophobic peptides: novel regulators within bacterial membrane. Mol Microbiol 72: 5–1110.1111/j.1365-2958.2009.06626.x. 19210615 10.1111/j.1365-2958.2009.06626.x

[6] Hemm MR, Paul BJ, Schneider TD, Storz G, Rudd KE (2008) Small membrane proteins found by comparative genomics and ribosome binding site models. Mol Microbiol 70: 1487–1501. 19121005 10.1111/j.1365-2958.2008.06495.x PMC2614699

[7] Hemm MR, Paul BJ, Miranda-Rios J, Zhang A, Soltanzad N, et al (2010) Small Stress Response Proteins in Escherichia coli: Proteins Missed by Classical Proteomic Studies. J Bacteriol 192: 46–58. 19734316 10.1128/JB.00872-09 PMC2798279

[8] Bishop RE, Leskiw BK, Hodges RS, Kay CM, Weiner JH (1998) The entericidin locus of Escherichia coli and its implications for programmed bacterial cell death. J Mol Biol 280: 583–596 doi:DOI: 10.1006/jmbi.1998.1894. 9677290 10.1006/jmbi.1998.1894

[9] Gassel M, Möllenkamp T, Puppe W, Altendorf K (1999) The KdpF Subunit Is Part of the K+-translocating Kdp Complex of Escherichia coli and Is Responsible for Stabilization of the Complex in Vitro. J Biol Chem 274: 37901–37907. 10608856 10.1074/jbc.274.53.37901

[10] Wong RS, McMurry LM, Levy SB (2000) ‘Intergenic’ blr gene in Escherichia coli encodes a 41-residue membrane protein affecting intrinsic susceptibility to certain inhibitors of peptidoglycan synthesis. Mol Microbiol 37: 364–370. 10931331 10.1046/j.1365-2958.2000.01998.x

[11] Cole C, Barber JD, Barton GJ (2008) The Jpred 3 secondary structure prediction server. Nucleic Acids Res 36: W197–W201. 18463136 10.1093/nar/gkn238 PMC2447793

[12] Weel-Sneve R, Bjoras M, Kristiansen KI (2008) Overexpression of the LexA-regulated tisAB RNA in E. coli inhibits SOS functions; implications for regulation of the SOS response. Nucleic Acids Res 36: 6249–6259. 18832374 10.1093/nar/gkn633 PMC2577331

[13] Wickens HJ, Pinney RJ, Mason DJ, Gant VA (2000) Flow Cytometric Investigation of Filamentation, Membrane Patency, and Membrane Potential in Escherichia coli following Ciprofloxacin Exposure. Antimicrob Agents Chemother 44: 682–687. 10681338 10.1128/aac.44.3.682-687.2000 PMC89746

[14] Odsbu I, Morigen, Skarstad K (2009) A Reduction in Ribonucleotide Reductase Activity Slows Down the Chromosome Replication Fork but Does Not Change Its Localization. PLoS ONE 4: e7617 doi:10.1371/journal.pone.0007617. 19898675 10.1371/journal.pone.0007617 PMC2773459

[15] Vogel J, Argaman L, Wagner EG, Altuvia S (2004) The small RNA IstR inhibits synthesis of an SOS-induced toxic peptide. Curr Biol 14: 2271–2276. 15620655 10.1016/j.cub.2004.12.003

[16] Darfeuille F, Unoson C, Vogel Jr, Wagner EG (2007) An Antisense RNA Inhibits Translation by Competing with Standby Ribosomes. Mol Cell 26: 381–392 doi:10.1016/j.molcel.2007.04.003. 17499044 10.1016/j.molcel.2007.04.003

[17] Kawano M, Oshima T, Kasai H, Mori H (2002) Molecular characterization of long direct repeat (LDR) sequences expressing a stable mRNA encoding for a 35-amino-acid cell-killing peptide and a cis-encoded small antisense RNA in Escherichia coli. Mol Microbiol 45: 333–349. 12123448 10.1046/j.1365-2958.2002.03042.x

[18] Eguchi Y, Itou J, Yamane M, Demizu R, Yamato F, Okada, et al (2007) B1500, a small membrane protein, connects the two-component systems EvgS/EvgA and PhoQ/PhoP in Escherichia coli. PNAS 104: 18712–18717. 17998538 10.1073/pnas.0705768104 PMC2141842

[19] Alix E, Blanc-Potard AB (2008) Peptide-assisted degradation of the Salmonella MgtC virulence factor. EMBO J 27: 546–557. 18200043 10.1038/sj.emboj.7601983 PMC2241655

[20] Unoson C, Wagner EG (2008) A small SOS-induced toxin is targeted against the inner membrane in Escherichia coli. Mol Microbiol 70: 258–270. 18761622 10.1111/j.1365-2958.2008.06416.x

[21] Levin-Zaidman S, Frenkiel-Krispin D, Shimoni E, Sabanay I, Wolf SG, Minsky A (2000) Ordered intracellular RecA-DNA assemblies: A potential site of in vivo RecA-mediated activities. Proc Natl Acad Sci U S A 97: 6791–6796. 10829063 10.1073/pnas.090532397 PMC18741

[22] Modell JW, Hopkins AC, Laub MT (2011) A DNA damage checkpoint in Caulobacter crescentus inhibits cell division through a direct interaction with FtsW. Genes & Development 25: 1328–1343. 21685367 10.1101/gad.2038911 PMC3127433

[23] Barbe J, Villaverde A, Cairo J, Guerrero R (1986) ATP hydrolysis during SOS induction in Escherichia coli. J Bacteriol 167: 1055–1057. 3528124 10.1128/jb.167.3.1055-1057.1986 PMC215980

[24] Kamensek S, Podlesek Z, Gillor O, Zgur-Bertok D (2010) Genes regulated by the Escherichia coli SOS repressor LexA exhibit heterogeneous expression. BMC Microbiol 10: 283 1471-2180-10-283 [pii];10.1186/1471-2180-10-283 [doi]. 21070632 10.1186/1471-2180-10-283 PMC2994835

[25] McCool JD, Long E, Petrosino JF, Sandler HA, Rosenberg SM, et al (2004) Measurement of SOS expression in individual Escherichia coli K-12 cells using fluorescence microscopy. Mol Microbiol 53: 1343–1357 10.1111/j.1365-2958.2004.04225.x [doi];MMI4225 [pii]. 15387814 10.1111/j.1365-2958.2004.04225.x

[26] Friedman N, Vardi S, Ronen M, Alon U, Stavans J (2005) Precise temporal modulation in the response of the SOS DNA repair network in individual bacteria. PLoS Biol 3: e238 doi:10.1371/journal.pbio.0030238. 15954802 10.1371/journal.pbio.0030238 PMC1151601

[27] Yamaguchi Y, Park JH, Inouye M (2011) Toxin-antitoxin systems in bacteria and archaea. Annu Rev Genet 45: 61–79 10.1146/annurev-genet-110410-132412 [doi]. 22060041 10.1146/annurev-genet-110410-132412

[28] Dörr T, Vuli-ç M, Lewis K (2010) Ciprofloxacin Causes Persister Formation by Inducing the TisB toxin in *Escherichia coli* . PLoS Biol 8: e1000317 doi:10.1371/journal.pbio.1000317. 20186264 10.1371/journal.pbio.1000317 PMC2826370

[29] Dewitt SK, Adelberg EA (1962) The Occurrence of a Genetic Transposition in a Strain of Escherichia Coli. Genetics 47: 577–585. 17248104 10.1093/genetics/47.5.577 PMC1210353

[30] Datsenko KA, Wanner BL (2000) One-step inactivation of chromosomal genes in Escherichia coli K-12 using PCR products. Proc Natl Acad Sci U S A 97: 6640–6645. 10829079 10.1073/pnas.120163297 PMC18686

[31] Wilson GG, Young KY, Edlin GJ, Konigsberg W (1979) High-frequency generalised transduction by bacteriophage T4. Nature 280: 80–82. 15305587 10.1038/280080a0

[32] Brosius J (1984) Plasmid vectors for the selection of promoters. Gene 27: 151–160 doi:DOI: 10.1016/0378-1119(84)90136-7. 6327464 10.1016/0378-1119(84)90136-7

[33] Seeberg E, Strike P (1976) Excision repair of ultraviolet-irradiated deoxyribonucleic acid in plasmolyzed cells of Escherichia coli. J Bacteriol 125: 787–795. 767327 10.1128/jb.125.3.787-795.1976 PMC236150

[34] Clark DJ, Maaløe O (1967) DNA replication and the division cycle in Escherichia coli. J Mol Biol 23: 99–112 doi:DOI: 10.1016/S0022-2836(67)80070-6.

