# Supplementary figures and images for: Single Transmembrane Peptide DinQ Modulates Membrane-Dependent Activities
Source: PLoS Genet. 2013 Feb 7;9(2):e1003260. doi: 10.1371/journal.pgen.1003260 (PMC3567139; doi:10.1371/journal.pgen.1003260)

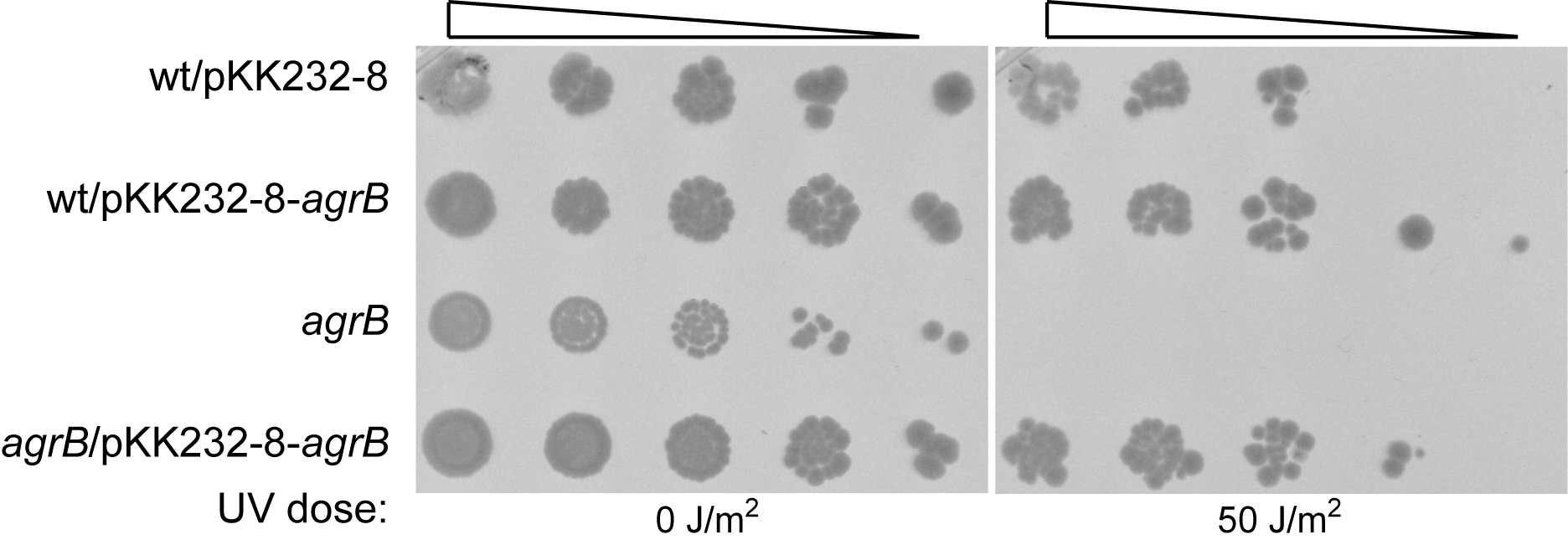

Supplement: Figure S1 — Complementation. Serially diluted (10−1–10−5cells ml−1) log phase cultures of wt (AB1157) or agrB (BK4043) with expression vector pKK232-8 or vector constructs with agrB (pKK232-8-agrB = pBK446) were spotted onto LB plates and exposed to 0 J/m2 UV (left panel) or 50 J/m2 UV (right panel). Pictures were taken 1 day after incubation at 37°C. (TIF) [file pgen.1003260.s001.tif]

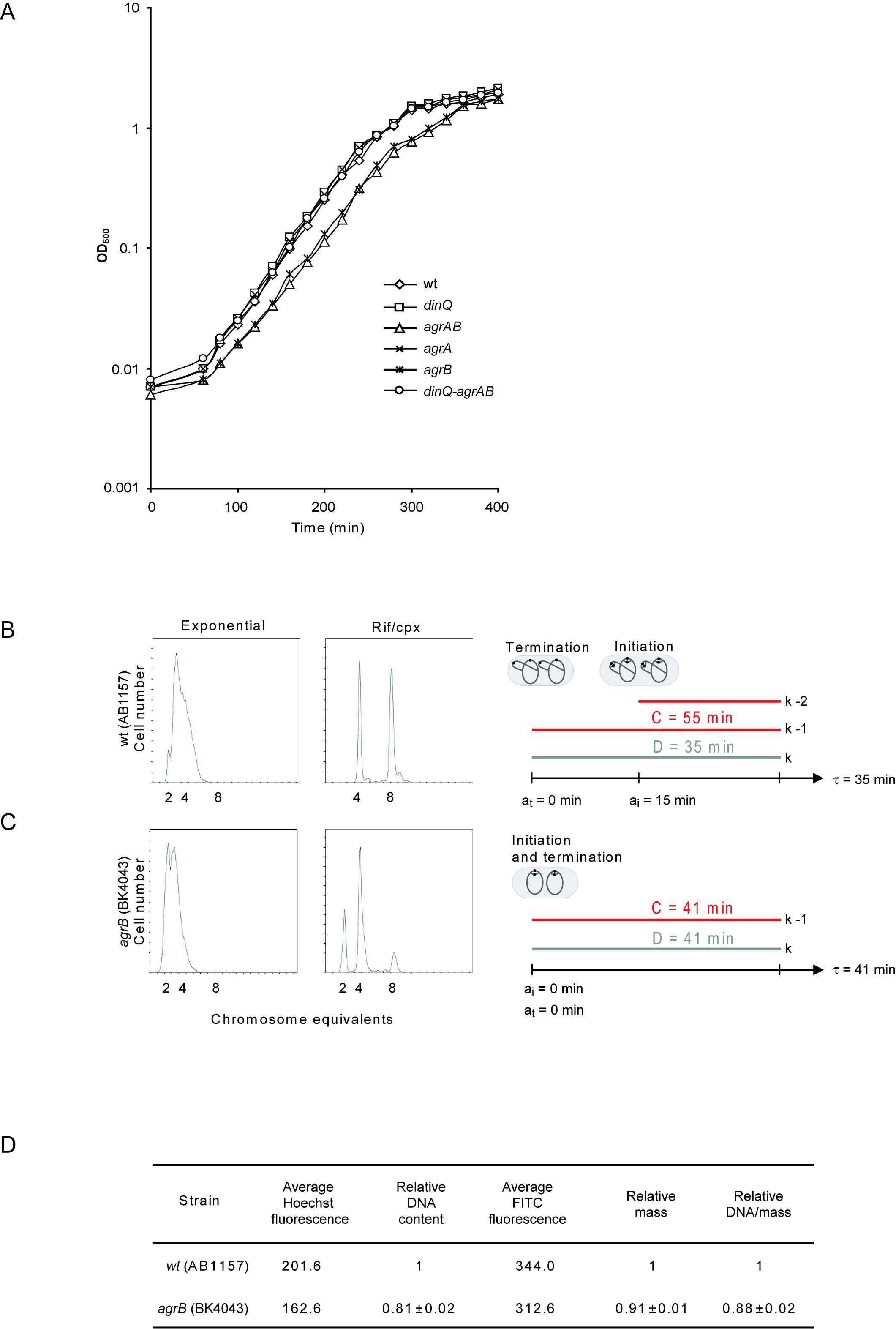

Supplement: Figure S2 — Growth curves, DNA histograms, and cell cycle analysis of wild-type cells and agrB mutant cells. (A) Growth rates of wt (AB1157, diamond) compared to dinQ (BK4040, square), agrAB (BK4041, triangle), agrA (BK4042, cross), agrB (BK4043, star) and dinQ-agrAB (BK4044, open circle). (B, C and D) Cell cycle analysis and flow cytometry of wt (AB1157) (B) and agrB (BK4043) (C). Cells were grown exponentially in AB minimal medium supplemented with 1 µg/ml thiamine, 0.2% glucose and 0.5% casamino acids at 37°C to OD450 = 0.15. Cells were either harvested or treated with 300 µg/ml rifampicin and 10 µg/ml cephalexin for 5 generations. The left and middle panels show DNA histograms of exponentially growing cells and cells treated with rifampicin (rif) and cephalexin (cpx), respectively, where the number of cells is plotted against the number of chromosome equivalents. The number of chromosome equivalents for rifampicin- and cephalexin-treated cells corresponds to the number of origins at the time of drug action. The right panel shows a schematic diagram of the cell cycle. The timeline at the bottom shows the generation time (τ) as well as the times for initiation (ai) and termination (at) of replication. Each horizontal bar represents one generation where the “current” generation is denoted k, the “mother” generation k-1 and the “grandmother” generation k-2. The C period (elongation) is colored red and the D period (segregation and cell division) is colored grey as one replication cycle is followed through three (AB1157, B) and two (BK4043, C) generations. Cells showing the replication pattern at the time of initiation and termination of replication are drawn on top of the diagram. A black dot and a grey circle represent the origin and the chromosome, respectively. The C+D period was determined from the initiation age, generation time and number of generations spanned by C+D. The initiation age and number of generations spanned were found from the rif/cpx histogram and the relat [file pgen.1003260.s002.tif]

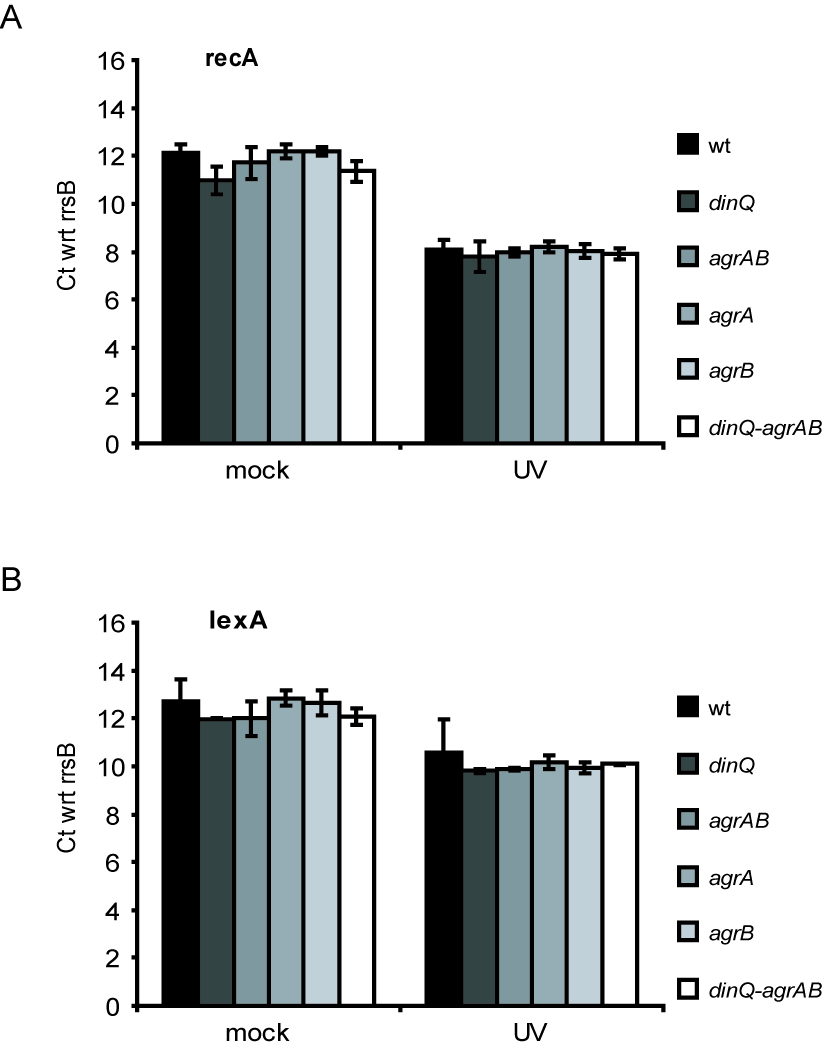

Supplement: Figure S3 — RT–qPCR showing stable recA and lexA mRNA concentrations with regards to rrsB in various dinQ regulon mutant strains both before and after UV exposure. The graphs show the mRNA concentrations of the SOS response regulators recA mRNA (A) and lexA mRNA (B) relative to the rrsB transcript before and after UV exposure. The relative concentrations in various mutant strains: dinQ (BK4040), agrAB (BK4041), agrA (BK4042), agrB (BK4043), dinQ-agrAB (BK4044) and the isogenic wt reference strain (AB1157) are compared. No significant difference was seen between the different strains indicating that the disrupted regions do not affect the induction of the SOS response at the transcription level. cDNA was synthesized from DNaseI treated total RNA (1 µg) using the High Capacity cDNA Reverse Transcription Kit (ABI) according to the manufactures' instructions. Power SYBR Green PCR MasterMix together with a StepOnePlus Real-time PCR System (ABI), cDNA (5 ng) and rrsB, recA or lexA primers (Table S2) to generate real-time plots that were automatically processed by the StepOne Software v2.0.1 to calculate cycle threshold (Ct) values. The primers were automatically selected using the Primer Express 3.0 software (ABI). Four independent samples were run in quadruplet to generate a mean Ct value relative to the rrsB (16S ribosomal RNA) transcript, ΔCt, which was used as the endogenous control. The ΔCt values fall after UV exposure due to the stable larger amount of rrsB transcript and a rising but reduced amount of recA or lexA transcript. (TIF) [file pgen.1003260.s003.tif]

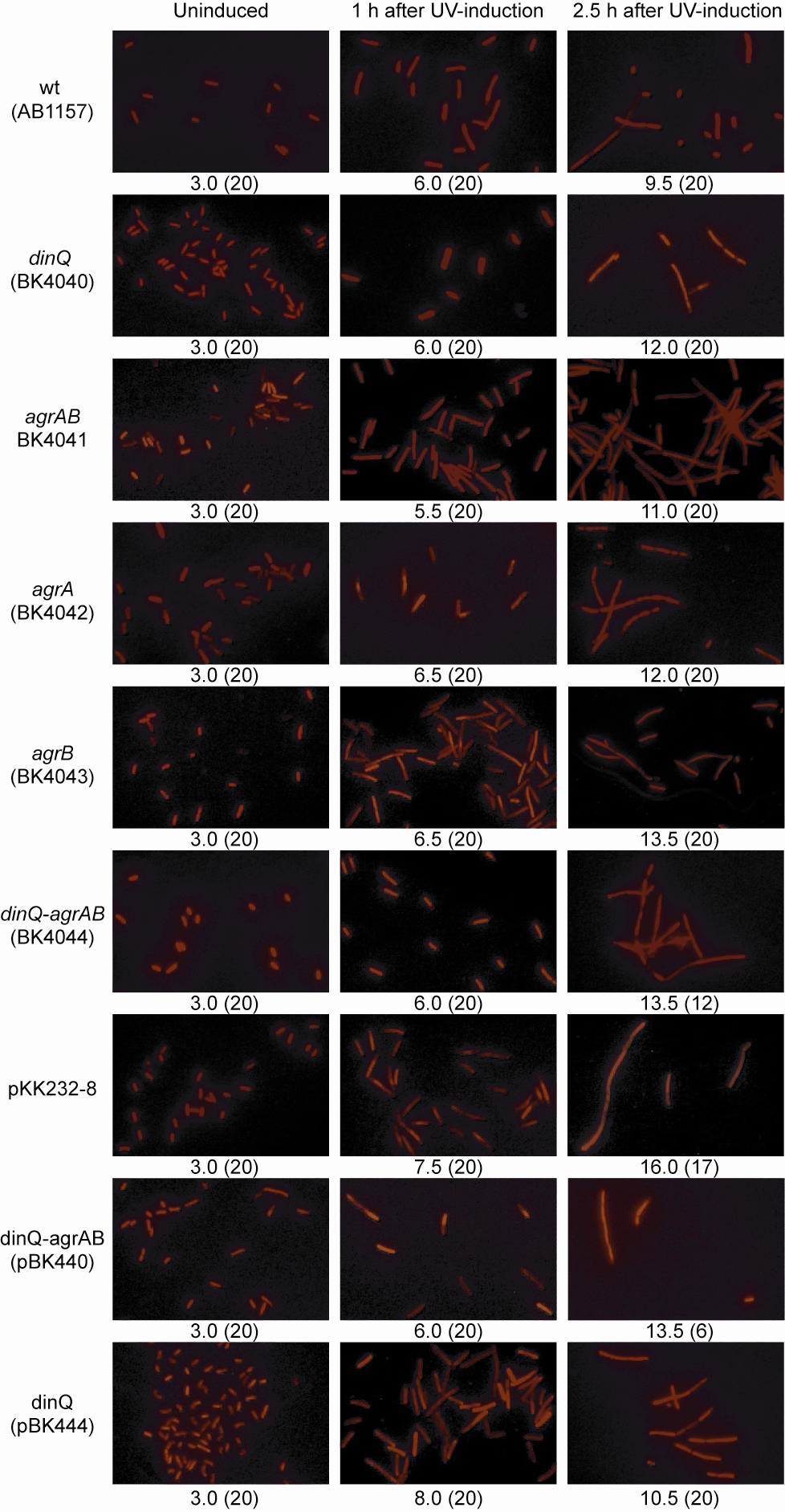

Supplement: Figure S4 — Filamentation. Fluorescence microscopy of UV exposed (50 J/m2) strains as indicated followed by growth for 1 and 2.5 h. Cells were grown in K-medium and stained with acridine orange. Each image is quantified and median values in µm are given together with number of cells measured (in parentheses). (TIF) [file pgen.1003260.s004.tif]

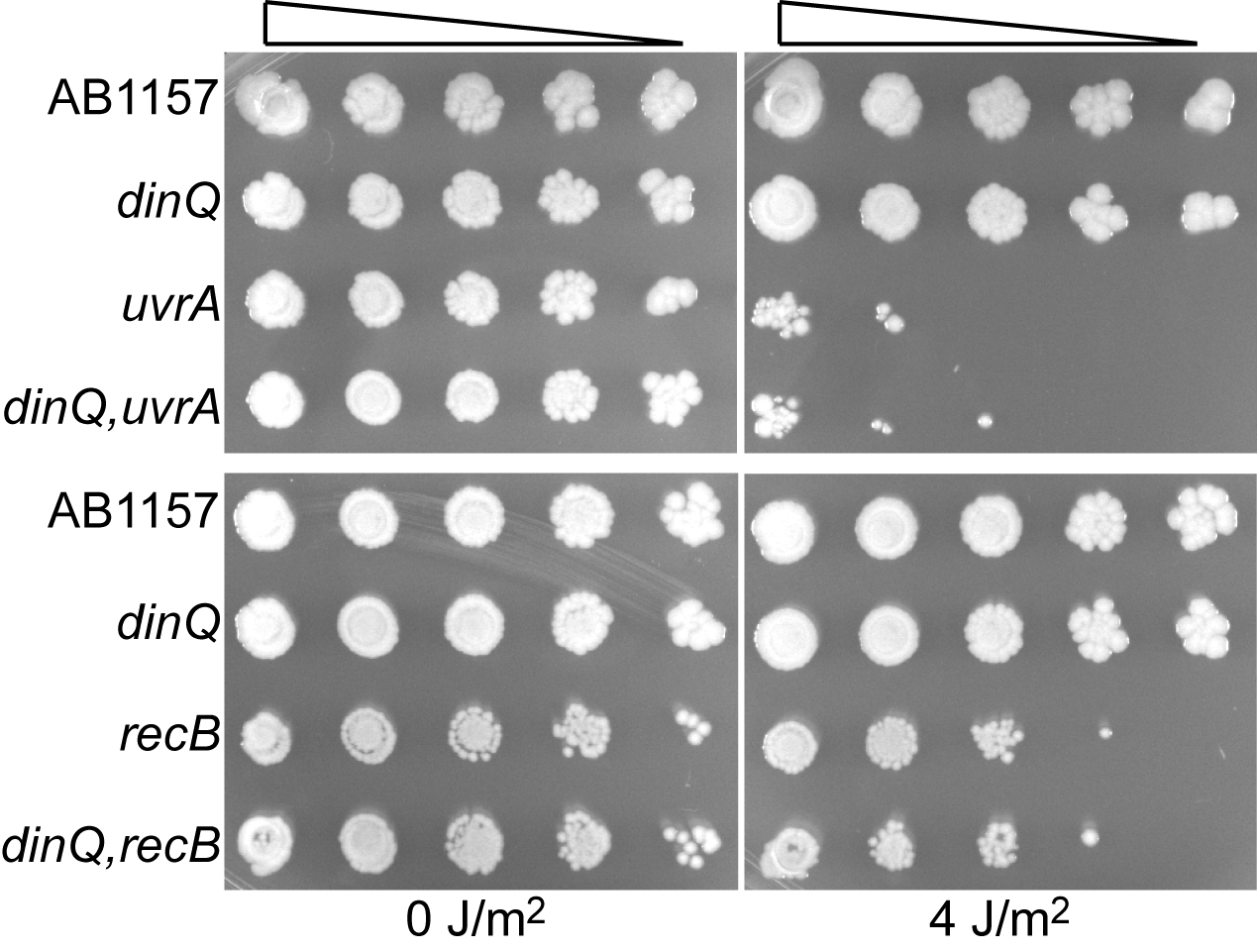

Supplement: Figure S5 — Survival of (dinQ,recB) and (dinQ, uvrA) mutants. Serially diluted Serially diluted (10−1–10−5cells ml−1) log phase cultures of wt (AB1157), ΔdinQ (BK4140), uvrA::kan (BK4180), ΔdinQ,uvrA (BK4141), recB::kan (BK4110) or ΔdinQ,recB::kan (BK4142) were spotted onto LB plates and exposed to 4 J/m2 (right panel), unexposed panels to the left. Pictures were taken 1 day after incubation at 37°C. (TIF) [file pgen.1003260.s005.tif]
