# Supplementary material for: Single Transmembrane Peptide DinQ Modulates Membrane-Dependent Activities
Source: PLoS Genet. 2013 Feb 7;9(2):e1003260. doi: 10.1371/journal.pgen.1003260 (PMC3567139; doi:10.1371/journal.pgen.1003260)
Supplement: Table S1 — Characteristics of bacterial strains and plasmids used in this study. (DOCX) [file pgen.1003260.s006.docx]

| Strain or plasmid | Description or genotype |
| --- | --- |
| AB1157 | F-, *thr-1*, *araC14*, *leuB6*(Am), *Δ(gpt-proA)62*, *lacY1*, *tsx-33*, *’sr’-0*, *glnV44*(AS), *galK2*(Oc), *&lambda^-^*, *Rac-0*, *hisG4*(Oc), *rfbC1*, *mgl-51*, *rpoS396*(Am), *rpsL31*(strR), *kdgK51*, *xylA5*, *mtl-1*, *argE3*(Oc), *thi-1* |
| BK4040 | AB1157 *dinQ::kan* (3645755…3645969) |
| BK4041 | AB1157 *agrAB::kan* (3646072…3646415) |
| BK4042 | AB1157 *agrA::kan* (3646072…3646185) |
| BK4043 | AB1157 *agrB::kan* (3646302…3646415) |
| BK4044 | AB1157 *dinQ-agrAB::kan* (3645755…3646415) |
| BK4110 | AB1157 *recB::kan* |
| BK4112 | AB1157 *ΔagrB recB::kan* |
| BK4148 | AB1157 *ΔagrB* |
| BK4180 | AB1157 *uvrA::kan* |
| BK4182 | AB1157 *ΔagrB uvrA::kan* |
| BK4183 | AB1157 *ΔuvrA recB::kan* |
| BW25113 | F-, *Δ(araD-araB)567*, *ΔlacZ4787*(::rrnB-3), *&lambda^-^*, *rph-1*, *Δ(rhaD-rhaB)568*, *hsdR514* |
| ER2566 | *F- λ- fhuA2 [lon] ompT lacZ::T7 gene 1 gal sulA11 Δ(mcrC-mrr)114::IS10 R(mcr-73::miniTn10-TetS)2 R(zgb-210::Tn10)(TetS) endA1 [dcm]* |
| ER2738 | F´*proA+B+ lacIq Δ(lacZ)M15 zzf::Tn10(TetR)/ fhuA2 glnV Δ(lac-proAB) thi-1 Δ(hsdS-mcrB)5* |
| BW7623 | Hfr(PO43), *purK79::Tn10*, *λ^-^*, *relA1*?, *spoT1*? |
| MG1655 | F-, *λ^-^*, *rph-1* |
| BK5444 | MG1655 *dinQ-agrAB::cat* (cat swap of dinQ-agrAB, primers 13931/  13921) |
| BK5300 | MG1655 wild type (*kan^r^*), SOEing fragments 1+2 |
| BK5342 | MG1655 *ΔagrB* (*kan^r^*), SOEing fragments 1+3 |
| BK5350 | MG1655 *dinQ*-K4stop, AAA to TAA of *dinQ* ORF5 (*kan^r^*)  SOEing fragments 1+4+5 |
| BK5352 | MG1655 *ΔagrB*, *dinQ*-K4stop (*kan^r^*), SOEing fragments 1+6+5 |
| BK5360 | MG1655 *dinQ*-A108T-C112G-A115T (*kan^r^*). SOEing fragments 1+7+8 |
| BK5362 | MG1655 *Δagr,* *dinQ*-A108T-C112G-A115T *(kan^r^),* SOEing fragments 1+9+8 |
| BK5370 | MG1655 DinQ-3xFLAG (kan^r^) |
| BK5372 | MG1655 *ΔagrB*, DinQ-3xFLAG (kan^r^) |
|  |  |
| pKK232-8 | Medium copy number cloning vector with a promoterless *cat* used for promoter selection. |
| pBK440 | pKK232-8 *dinQ-agrAB* locus inserted in the *BamHI* restriction site |
| pBK444 | pKK232-8 *dinQ* inserted in the *BamHI* restriction site |
| pBK446 | pKK232-8 *agrB* inserted in the *BamHI* restriction site |
| pKD46 | Lambda Red recombinase expression plasmid, derivative of pINT-ts which contain *araC*-*P_araB_* and γ β *exo* |
| pET28b(+) | Bacterial expression vector with T7 and LacO promoters, adds T7 and 6×His tags, includes LacI gene; kanamycin restistance; CpoI-based in-frame, single-cut directional cloning. |
| pET28b(+)-DinQ I | pET28b(+) 18 aa ORF1 of *dinQ* inserted in the *NcoI*-*BamHI* restriction sites |
| pET28b(+)-DinQ II | pET28b(+) 49 aa ORF2 of *dinQ* inserted in the *NcoI*-*BamHI* restriction sites |
| pET28b(+)-DinQ III | pET28b(+) 42 aa ORF3 of *dinQ* inserted in the *NcoI*-*BamHI* restriction sites |
| pET28b(+)-DinQ IV | pET28b(+) 38 aa ORF4 of *dinQ* inserted in the *NcoI*-*BamHI* restriction sites |
| pET28b(+)-DinQ V | pET28b(+) 27 aa ORF5 of *dinQ* inserted in the *NcoI*-*BamHI* restriction sites |
| pET28b(+)-3×FLAG-DinQ V | pET28b(+) 3×FLAG sequence N-terminal of 27 aa ORF5 of *dinQ* inserted in the *NcoI*-*BamHI* restriction sites |
