# Supplementary material for: Single Transmembrane Peptide DinQ Modulates Membrane-Dependent Activities
Source: PLoS Genet. 2013 Feb 7;9(2):e1003260. doi: 10.1371/journal.pgen.1003260 (PMC3567139; doi:10.1371/journal.pgen.1003260)
Supplement: Table S2 — Transformation efficiency. Exponentially growing cells of wt (AB1157) and agrB (BK4043/BK4180) were transformed by electroporation with plasmids pKK232-8 and pBK444 (dinQ). Dilutions were spread on LB plates and transformed cells were counted. (DOCX) [file pgen.1003260.s007.docx]

| ID | Description | Sequence |
| --- | --- | --- |
| 1474/1475 | pBK440 | ACGCTGGATCCGGAAGATGGTC / CGTCGGGATCCAGACGATATTCC |
| 3394/3395 | pBK444 | CGCGGATCCATACTGAAACTATTGCAAG / GGCAGAAAGCTTCGTGACAATGCGTTA |
| 2082/2083 | BK4040 | TACGTTTCCGCTATCAGTTCAGAAGCTGAAGCAGAAAGCGGTGTAGGCTGGAGCTGCT / ATCCAGTTAGCTCTGAGGCATTTTCACTCTGGCAATGCGCATGAATATCCTCCTTAGTTCC |
| 2084/2085 | BK4041 | GTTATAAAACCAGCACGTCCTTGCAATAGTTTCAGTATGGTATGGACAGCAAGCGAAC / AATAGGTGTGAATTTTGAGTTGGCTATTCATTTGAAAGGAGAAGAACTCCAGCATGAG |
| 2084/2182 | BK4042 | GTTATAAAACCAGCACGTCCTTGCAATAGTTTCAGTATGGTATGGACAGCAAGCGAAC / GATTTATATACTTTCACTGGGTCATCGTCATATTAAGCCTGAAGAACTCCAGCATGAG |
| 2183/2085 | BK4043 | GCTGAAGAAATCGCTAATTCTTGCAATGTTAGCCACTGGCTATGGACAGCAAGCGAAC / AATAGGTGTGAATTTTGAGTTGGCTATTCATTTGAAAGGAGAAGAACTCCAGCATGAG |
| 2082/2085 | BK4044 | TACGTTTCCGCTATCAGTTCAGAAGCTGAAGCAGAAAGCGGTGTAGGCTGGAGCTGCT / AATAGGTGTGAATTTTGAGTTGGCTATTCATTTGAAAGGAGAAGAACTCCAGCATGAG |
| 7891/7892 | pET28b(+)-DinQ I | CATCCATGGATGCGCATAAACGCTTTCAA / CGCGGATCCTCAGTTAACGGCACCACCC |
| 4999/5000 | pET28b(+)-DinQ II | CATCCATGGATGCGCCGTGACAGGCAGTG / CGCGGATCCTCAGTTCAGAAGCTGAAGC |
| 7894/4999 | pET28b(+)-DinQ III | CATCCATGGATGAGTAAGCGGATGCATTC / CGCGGATCCTCAGTTCAGAAGCTGAAGC |
| 7897/4999 | pET28b(+)-DinQ IV | CATCCATGGATGCATTCTCACTCCATCGC / CGCGGATCCTCAGTTCAGAAGCTGAAGC |
| 9068/4999 | pET28b(+)-DinQ V | CATCCATGG**TT**GATAAAGCAATCATCGTTC / CGCGGATCCTCAGTTCAGAAGCTGAAGC |
| 6291/6292 | Riboprobe agrA/B | TAATACGACTCACTATAGGAAACCGACTTTGCGTCGG / TCTCACTCCAGCCAGAGC |
| 4980/4981 | Riboprobe dinQ T7 | TAATACGACTCACTATAGGTACGTTTCCGCTATCAGTTC / GCTCTGAGGCATTTTCACTC |
| 4994 | Primer extension agrA/B | CTTTCCAGCCCTGAGTTGG |
| 4995 | Primer extension dinQ | GAATGCATCCGCTTACTCATCCAC |
| 5827/5828 | recB PCR | CAGCCGTGGTTCTACAATC / GCTCCACAGCTTCCAGTAAT |
| 10809/  10810 | uvrB PCR | GCGGAGTTTACGCTGTATC / GCCAGCGATATTCCGTATC |
| 8678/8679 | RT-qPCR recA | TGGTTGACCTGGGCGTAAAA / CTGACCGATCTTCTCACCTTTGT |
| 8445/8446 | RT-qPCR lexA | AGAAGGGTTGCCGCTGGTA / TTGCGCCAGAAGTGGTTCA |
| 8289/8288 | RT-qPCR rrsB | CCGCTGGCAACAAAGGATAA / CGTGTTGTGAAATGTTGGGTTAA |
| 12615/  12614 | pCR2.1-DinQ-3xFLAG | GATTATAAAGATCATGATATCGATTACAAGGATGACGATGACAAGATTGATAAAGCAATCATC / CGATATCATGATCTTTATAATCACCGTCATGGTCTTTGTAGTCCACCCGTTTTCTCCATGC |
| 13931/  13921 | cat swap of dinQ-agrAB.  Template pKD3 | TCTTAGCCCTTAATTACGTTTCCGCTATCAGTTCAGAAGCTGAAGCTGTGACGGAAGATCACT / GATAAGTCAAAAACATATATGACTTAACGAATGTGTAAGTGCAGAGGAGAACTTCATTTAAATGGC |
| 13732/  13731 | SOEing fragment 1 kan cassette Template pKD4 | GATAAGTCAAAAACATATATGACTTAACGAATGTGTAAGTGCAGAGGACATATGAATATCCTCCTTAGT / GACCTCCTTTCAAATGAATAGCCAACTCAAAATTCACACCTATTACCTGTGTAGGCTGGAGCTGCTTC |
| 13829/  13825 | SOEing fragment 2  Template pBK440 | GGCTATTCATTTGAAAGGAG / TTCGTGACAATGCGTTAAATG |
| 13833/  13825 | SOEing fragment 3  Template pBK440 | GGCTATTCATTTGAAAGGAGGTCAATTAGCGATTTCTTCAGCTG / TTCGTGACAATGCGTTAAATG |
| 13829/  13832 | SOEing fragment 4  Template pBK440 | GGCTATTCATTTGAAAGGAG / GAACGATGATTGCTTAATCAATCACCCGTTT |
| 13831/  13825 | SOEing fragment 5  Template pBK440 | AAACGGGTGATTGATTAAGCAATCATCGTTC / TTCGTGACAATGCGTTAAATG |
| 13833/  13832 | SOEing fragment 6  Template pBK440 | GGCTATTCATTTGAAAGGAGGTCAATTAGCGATTTCTTCAGCTG / GAACGATGATTGCTTAATCAATCACCCGTTT |
| 13829/  13837 | SOEing fragment 7  Template pBK440 | GGCTATTCATTTGAAAGGAG / AGAAAACAACACTCTCACTCCGGCCAGAGCATCAG |
| 13836/  13825 | SOEing fragment 8  Template pBK440 | GTGAGAGTGTTGTTTTCTAACAATGAGACATGC / TTCGTGACAATGCGTTAAATG |
| 13833/  13837 | SOEing fragment 9  Template pBK440 | GGCTATTCATTTGAAAGGAGGTCAATTAGCGATTTCTTCAGCTG / AGAAAACAACACTCTCACTCCGGCCAGAGCATCAG |
| 13829/  13828 | SOEing fragment 10  Template pBK440 | GGCTATTCATTTGAAAGGAG / CCTTGCAATAGTTTCAGTATG |
| 13827/  13825 | SOEing fragment 11  Template pCR2.1-DinQ-3xFLAG | CCATACTGAAACTATTGCAAG / TTCGTGACAATGCGTTAAATG |
| 13833/  13828 | SOEing fragment 12  Template pBK440 | GGCTATTCATTTGAAAGGAGGTCAATTAGCGATTTCTTCAGCTG / CCTTGCAATAGTTTCAGTATG |
| 12615/  12614 | pCR2.1-DinQ-3xFLAG | GATTATAAAGATCATGATATCGATTACAAGGATGACGATGACAAGATTGATAAAGCAATCATC / CGATATCATGATCTTTATAATCACCGTCATGGTCTTTGTAGTCCACCCGTTTTCTCCATGC |
