# Supplementary material for: Single Transmembrane Peptide DinQ Modulates Membrane-Dependent Activities
Source: PLoS Genet. 2013 Feb 7;9(2):e1003260. doi: 10.1371/journal.pgen.1003260 (PMC3567139; doi:10.1371/journal.pgen.1003260)
Supplement: Table S3 — Oligonucleotides used in this study. (DOCX) [file pgen.1003260.s008.docx]

| Transformants pr 10^8^ cells | AB1157 | BK4043 |
| --- | --- | --- |
| pKK232-8 | 38889 | 15420 |
| pBK444 | 254 | 0 |
